# Cooperative dihydrogen activation with unsupported uranium–metal bonds and characterization of a terminal U(iv) hydride†

**DOI:** 10.1039/d3sc04857h

**Published:** 2023-10-16

**Authors:** Robert J. Ward, Pokpong Rungthanaphatsophon, Patrick Huang, Steven P. Kelley, Justin R. Walensky

**Affiliations:** a Department of Chemistry, University of Missouri Columbia MO 65211 USA walenskyj@missouri.edu; b Department of Chemistry & Biochemistry, California State University East Bay Hayward CA 94542 USA

## Abstract

Cooperative chemistry between two or more metal centres can show enhanced reactivity compared to the monometallic fragments. Given the paucity of actinide–metal bonds, especially those with group 13, we targeted uranium(iii)–aluminum(i) and –gallium(i) complexes as we envisioned the low-valent oxidation state of both metals would lead to novel, cooperative reactivity. Herein, we report the molecular structure of [(C_5_Me_5_)_2_(MesO)U-E(C_5_Me_5_)], E = Al, Ga, Mes = 2,4,6-Me_3_C_6_H_2_, and their reactivity with dihydrogen. The reaction of H_2_ with the U(iii)–Al(i) complex affords a trihydroaluminate complex, [(C_5_Me_5_)_2_(MesO)U(μ_2_-(H)_3_)–Al(C_5_Me_5_)] through a formal three-electron metal-based reduction, with concomitant formation of a terminal U(iv) hydride, [(C_5_Me_5_)_2_(MesO)U(H)]. Noteworthy is that neither U(iii) complexes nor [(C_5_Me_5_)Al]_4_ are capable of reducing dihydrogen on their own. To make the terminal hydride in higher yields, the reaction of [(C_5_Me_5_)_2_(MesO)U(THF)] with half an equivalent of diethylzinc generates [(C_5_Me_5_)_2_(MesO)U(CH_2_CH_3_)] or treatment of [(C_5_Me_5_)_2_U(i)(Me)] with KOMes forms [(C_5_Me_5_)_2_(MesO)U(CH_3_)], which followed by hydrogenation with either complex cleanly affords [(C_5_Me_5_)_2_(MesO)U(H)]. All complexes have been characterized by spectroscopic and structural methods and are rare examples of cooperative chemistry in f element chemistry, dihydrogen activation, and stable, terminal ethyl and hydride compounds with an f element.

## Abstract 2

The cooperation between U(iii) and Al(i) centres leads to dihydrogen reduction.

## Graphical abstract

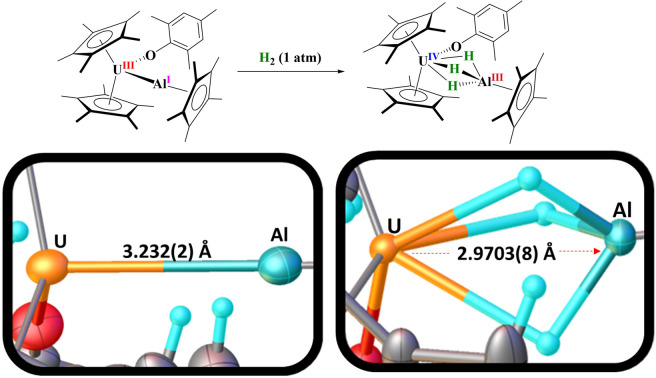

## Introduction

Cooperative chemistry^1^ between heterobimetallic complexes has attracted attention for years as they demonstrate enhanced and unique reactivity compared to monometallic complexes or their fragments alone.^2–7^ This has traditionally been done with an early transition metal, redox-inactive but highly Lewis acidic metal such as zirconium(iv) paired with a low-valent middle or late transition metal such as cobalt,^8–10^ iron,^11^ or iridium.^12^ While this reactivity is well-established with transition metals,^13^ the focus with f elements has been on the synthesis of new f element-metal bonds,^14–45^ with very little reactivity demonstrated with these complexes.^46,47^ In fact, reports of metal–metal cooperativity in f element chemistry are rare,^48^ while there are examples of using transition metals to enhance or form unusual moieties with f elements.^40,49,50^

Group 13 compounds bonded to f elements are rare,^51–63^ especially with the actinides. In fact, only one actinide–boron interaction with a 1,4-diborabenzene ligand,^64^ one uranium–aluminium bond,^14^ and two uranium–gallium bonds^15,43^ have been previously reported. Recently, our group has been interested in the interaction of low-valent aluminium with high-valent uranium and reported the reduction of a U(vi) bis-(imido) complex, [(C_5_Me_5_)_2_U{=N(4-^i^PrOC_6_H_4_)}_2_], with [Al(C_5_Me_5_)]_4_ to yield a U(iv)/Al(iii) heterobimetallic complex, [(C_5_Me_5_)_2_U{μ_2_-{N(4-^i^PrOC_6_H_4_)}_2_}Al(C_5_Me_5_)].^65^ The complex revealed a short U–Al distance of 3.071 Å, but no molecular orbital containing a metal–metal bond was found. Subsequently, we reported the uranium(iii) complex heteroleptic ligand framework, [(C_5_Me_5_)_2_(MesO)U(THF)], 1, Mes = 2,4,6-Me_3_C_6_H_2_,^66^ and have been investigating the reactivity of this complex with small molecules.^67,68^ We surmised the addition of [E(C_5_Me_5_)]_*n*_, E = Al, *n* = 4, Ga, *n* = 1, given their strong Lewis basicity, could produce a metal–metal bonded species by displacement of the THF solvent molecule and lead to an unsupported U–E bond, and the combination of these low-valent fragments would lead to more enhanced reactivity.

Herein, we report the successful conclusion to our hypothesis of forming both U–Al, 2, and U–Ga, 3, bonds from 1. In determining substrates to examine the reactivity of 2 and 3, cooperative chemistry has been previously shown with dihydrogen activation with heterobimetallic transition metal and main group complexes.^69–77^ We demonstrate that the U–E (E = Al, Ga) bonds reported here can activate dihydrogen, which shows cooperativity as neither U(iii) complexes nor [(C_5_Me_5_)Al]_4_ reduce H_2_ alone. In the case of U–Al, a trihydroaluminate is formed as well as a mononuclear U(iv) hydride complex, which occurs due to the cooperation of both uranium(iii) and aluminum(i) working in concert to reduce dihydrogen in a formally three-electron reduction. In the case of U–Ga, only the U(iv) hydride is observed. Mechanistic investigations show that the U(iv) hydride is mostly likely formed through hydrogenation of a ‘tuck-in’ species, and not through reduction by U(iii). Finally, the characterization of the rare, kinetically and thermally stable, terminal U(iv) hydride complex is reported through more efficient syntheses.

## Results and discussion

**Fig. 1:**
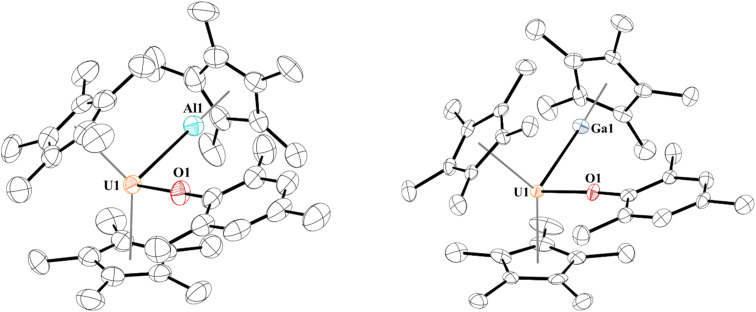
Thermal ellipsoid plot of 2 (left) and 3 (right) shown at the 50% probability level. The hydrogen atoms have been omitted for clarity.

When 1 is reacted with 0.25 equiv. of [Al(C_5_Me_5_)]_4_ at 60 °C or one equiv. of [Ga(C_5_Me_5_)] in toluene at room temperature, coordination was observed, leading to complexes with uranium–aluminium or –gallium bonds, [(C_5_Me_5_)_2_(MesO)UE(C_5_Me_5_)], E = Al, 2; Ga, 3. Heating of [Al(C_5_Me_5_)]_4_ is known to break up the tetramer to form the monomeric species in solution. We note that the aryloxide complex, [(C_5_Me_5_)_2_(2,6-^*t*^Bu_2_-4-MeC_6_H_2_O)U],^78^ does not react with [Al(C_5_Me_5_)]_4_ (at 60 °C) or [Ga(C_5_Me_5_)] after stirring overnight, leaving only starting material present in the ^1^H NMR spectrum, presumably due to insufficient access to the metal centre from the steric bulk of the aryloxide ligand. Dark brown-green, for 2, and black-green, for 3, crystals, suitable for X-ray crystallographic analysis, Fig. 1, were obtained from a saturated pentane solution at −14 °C.

Two unique molecules are in each unit cell of complex 2. In each molecule, the geometry about the uranium centre is pseudo-tetrahedral. One molecule has the two (C_5_Me_5_)^1−^ ligands eclipsed and has a U–Al distance of 3.232(2) Å, while the other molecule has staggered (C_5_Me_5_)^1−^ ligands and a U–Al distance of 3.201(3) Å. This is slightly longer than the sum of the covalent radii of U and Al of 3.17 Å.^79^ The U–O bond distances are 2.179(5) Å and 2.169(7) Å, and the U–O–C(*ipso*) angles of 176.4(5)° or 177.9(9)°, respectively. While close to linear, U–O–C(*ipso*) bond angles in uranium(iii) aryloxides are typically found in the 160–170° range.^80^ The Al–U–O angle is 100.37(15)° and 96.7(3)° in 2a and 2b, respectively, and we observe no interaction between the Al1 and O1 with a distance of 4.072(9) Å in 2a and 4.211(6) Å in 2b.

The ^1^H NMR spectrum for 2 shows two equivalent (C_5_Me_5_)^1−^ resonances at −12.96 ppm, which is further upfield than observed for many [(C_5_Me_5_)_2_U(X)(L)]^+^, X = monoanionic ligand, L = neutral donor ligand, complexes. For example, complex 1 has a (C_5_Me_5_)^1−^ resonance at −4.03 ppm, [(C_5_Me_5_)_2_(4-^*t*^BuC_6_H_4_O)U(THF)] is observed at −7.72 ppm, (C_5_Me_5_)_2_U(i)(THF) is at −1.09 ppm.^81^ These signals are similar to the −12.87 ppm in (C_5_Me_5_)_2_U(hpp), hpp = 1,3,4,6,7,8-hexahydro-2*H*-pyrimido[1,2-*a*]pyrimidinato.^82^ The (C_5_Me_5_)^1−^ resonance on Al(i) was located at −10.45 ppm which is upfield compared to [(C_5_H_4_SiMe_3_)_3_UAl(C_5_Me_5_)] at −6.89 ppm.^14^ The *ortho*-methyl groups split in two singlets found at −14.45 and −27.09 ppm, and the *para*-methyl group of the mesityl substituent appears at +4.31 ppm. These resonances are similar to those found in 1 with the *ortho*- and *para*-methyl groups observed at −16.00 ppm and +2.97 ppm, respectively. Furthermore, the *meta*-hydrogens also split in 2 into two singlets at 7.54 and 10.79 ppm. This indicates restricted rotation of the mesityl group in solution due to (C_5_Me_5_)Al coordination.

In 3, the geometry is also best described as pseudo-tetrahedral. The U–Ga bond of 3.1709(9) Å in 3 is at the limit of the sum of the covalent radii of U and Ga of 3.18 Å.^79^ The U–Ga bond is longer than the 3.0648(12) Å in [(C_5_H_4_SiMe_3_)_3_UGa(C_5_Me_5_)],^15^ but shorter than U–Ga bonds of 3.2115(8) and 3.2983(9) Å in the two molecules obtained in the asymmetric unit of a single crystal of [(TREN^TMS^)U{Ga(NArCH)_2_(THF)}], TREN^TMS^ = [N(CH_2_CH_2_NSiMe_3_)_3_], Ar = 2,6-^i^Pr_2_C_6_H_3_.^43^ Commensurate with the shorter U–Ga bond distance, the O–U–Ga angle is more acute at 95.97(11)°. The Ga1–O1 distance is also quite long at 4.022(4) Å, hence no interaction is taking place.

The difference in U–E bond distances can be traced to their respective E-centroid distances. In 2a and 2b the distances are indistinguishable at 1.895 and 1.899 Å, respectively, while the Ga-centroid distance in 3 is ∼0.1 Å longer at 1.984 Å. Because of this longer E-centroid distance in 3, the (C_5_Me_5_)^1−^ group on (C_5_Me_5_)Ga has greater flexibility in positioning itself towards the uranium centre. Therefore, a shorter bond distance is observed in 3, despite Ga(i) being a weaker Lewis base than Al(i).

At room temperature, no evidence of an equilibrium between 1 and [(C_5_Me_5_)Ga] is observed, and only 3 is found in solution. The two (C_5_Me_5_)^1−^ coordinated to uranium have resonances at −11.02 ppm as a broad singlet, while the (C_5_Me_5_)^1−^ for Ga(i) appears at −5.64 ppm. In addition, the mesityl ring is symmetric with only one resonance for the *ortho*-methyl groups at −18.76 ppm, the *para*-methyl group at +3.86 ppm, and one for the *meta*-hydrogens at +8.53 ppm. However, when we performed an NMR titration experiment as done with [(Me_3_SiC_5_H_4_)_3_U] and [(C_5_Me_5_)Ga], an equilibrium is observed over all temperatures examined (213–333 K), Fig. S7,† with a coalescence temperature around 260 K. This should allow us to extract thermodynamic data based on the chemical shift measurements, but the data obtained did not align with previous literature reported values for the enthalpy and entropy, so this system is more complicated than previous homoleptic systems examined. However, the qualitative observations that a soft donor such as [(C_5_Me_5_)Ga] outcompetes THF is unexpected, as well as only two equivalents of [(C_5_Me_5_)Ga] are needed to form the metal–metal bonded complex *versus* 10 equivalents with [(Me_3_SiC_5_H_4_)_3_U] indicates the unique features of this ligand environment.

**Fig. 2:**
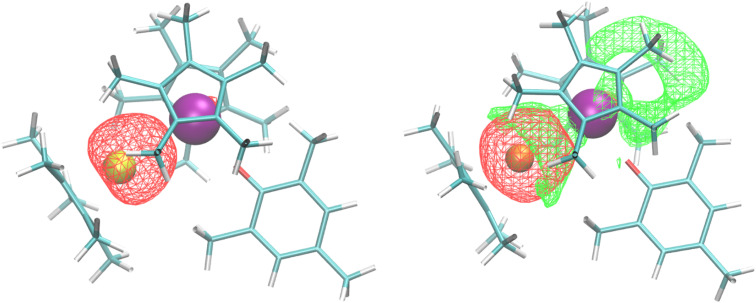
(Left) U–Al dative bond from NBO analysis and (Right) Ga 4s lone pair (red isosurface) donating to an empty U valence orbital (green isosurface).

Bonding analysis of both 2 and 3 were examined using density functional theory. For the U–Al complex, the calculated U–O distance of 2.17 Å is identical to the crystal structure, while the U–Al distance of 3.202 Å, compares well with the experimentally determined lengths of 3.232(2) or 3.201(3) Å in 2. The Natural Bond Orbital (NBO) analysis found a dative U–Al bond (Fig. 2) that is 20% uranium sd-character and 80% aluminium sp-character, with a Mayer bond order of 0.58. No other bonding NBOs involving the uranium were seen in the U–Al complex. The U–Ga complex has a similar calculated U–O distance, also at 2.17 Å, and a U–Ga distance of 3.188 Å, similar to the experimental length of 3.1709(9) Å in 3. However, the NBO analysis for the U–Ga complex did not find any bonding NBOs involving uranium. Instead, we find a lone pair NBO on the gallium that is primarily Ga 4s, shown in Fig. 2 (red isosurface). Note that NBOs correspond to Lewis-like bonds and lone pairs. One way to assess the role non-Lewis contributions to the bonding is to estimate donor–acceptor interactions *via* second-order perturbation analysis in the NBO basis. We find that the dominant contribution to the second-order energy is due to the donation of the lone pair NBO on gallium to a lone valence NBO on uranium (*E*_2_ = 64 kcal mol^−1^), shown in Fig. 2 (green isosurface). This uranium lone valence NBO is relatively delocalized and extends over the cavity region created by the (C_5_Me_5_)^1−^ and aryloxide ligands.

### Reactivity with H_2_

**Scheme 1:**
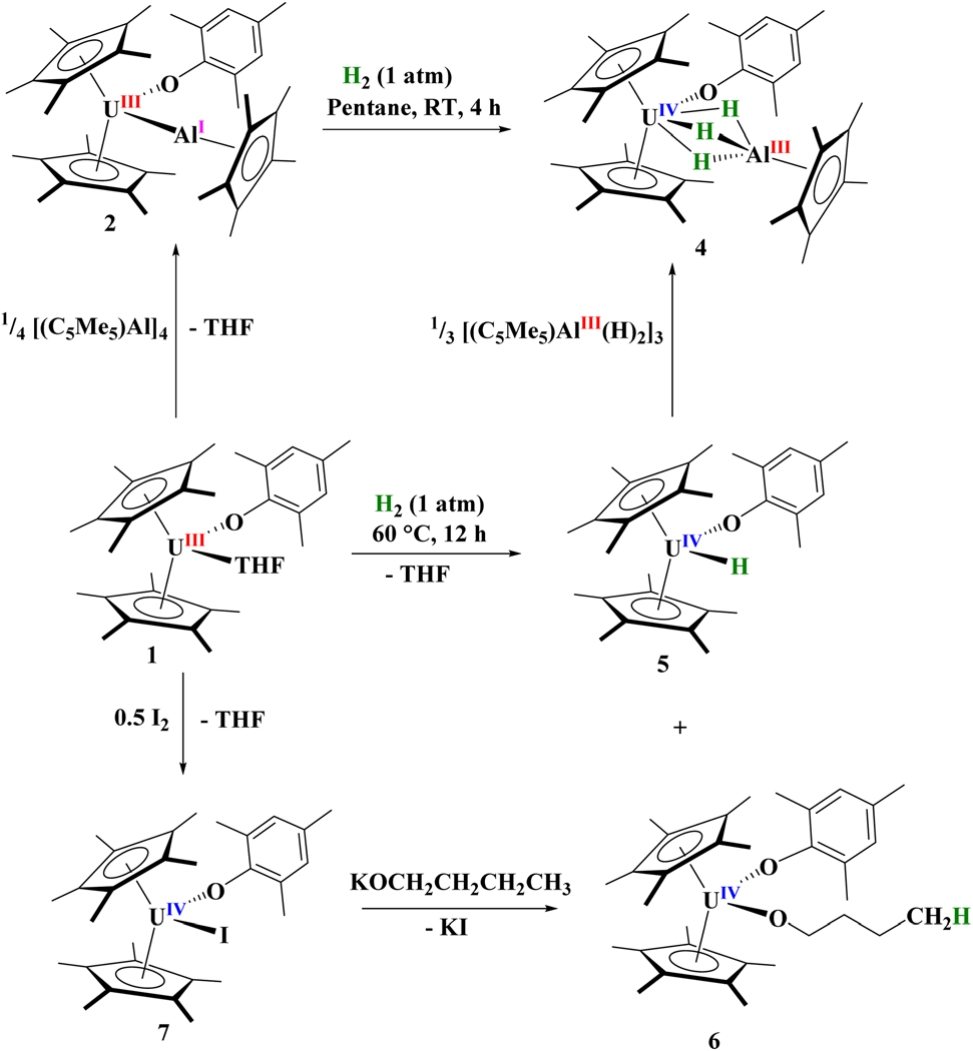
Formation of complexes 2–7.

**Scheme 2:**
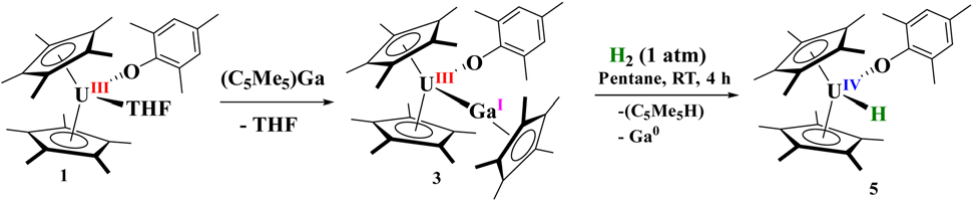
Reaction of 1 with [(C_5_Me_5_)Ga] and the resulting complex, 3, with H_2_.

**Fig. 3:**
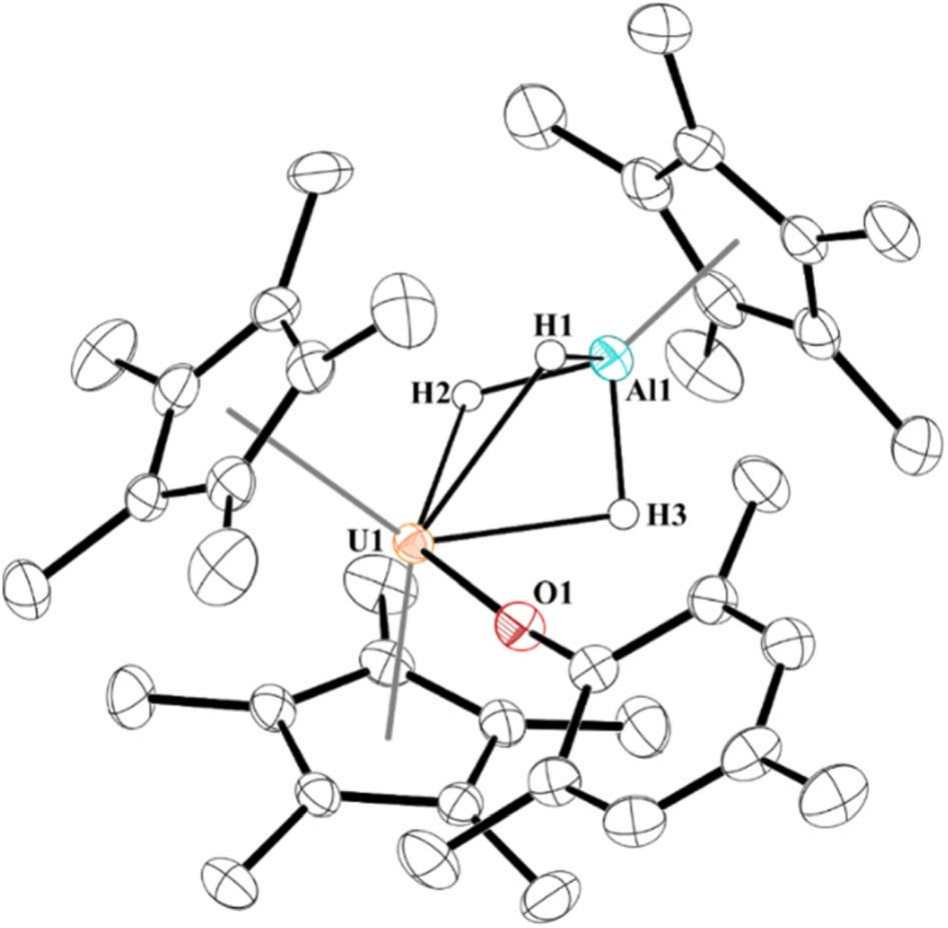
Thermal ellipsoid plot of 4 shown at the 50% probability level. Only the hydrogen atoms bridging between U1 and Al1 are shown for clarity.

**Scheme 3:**
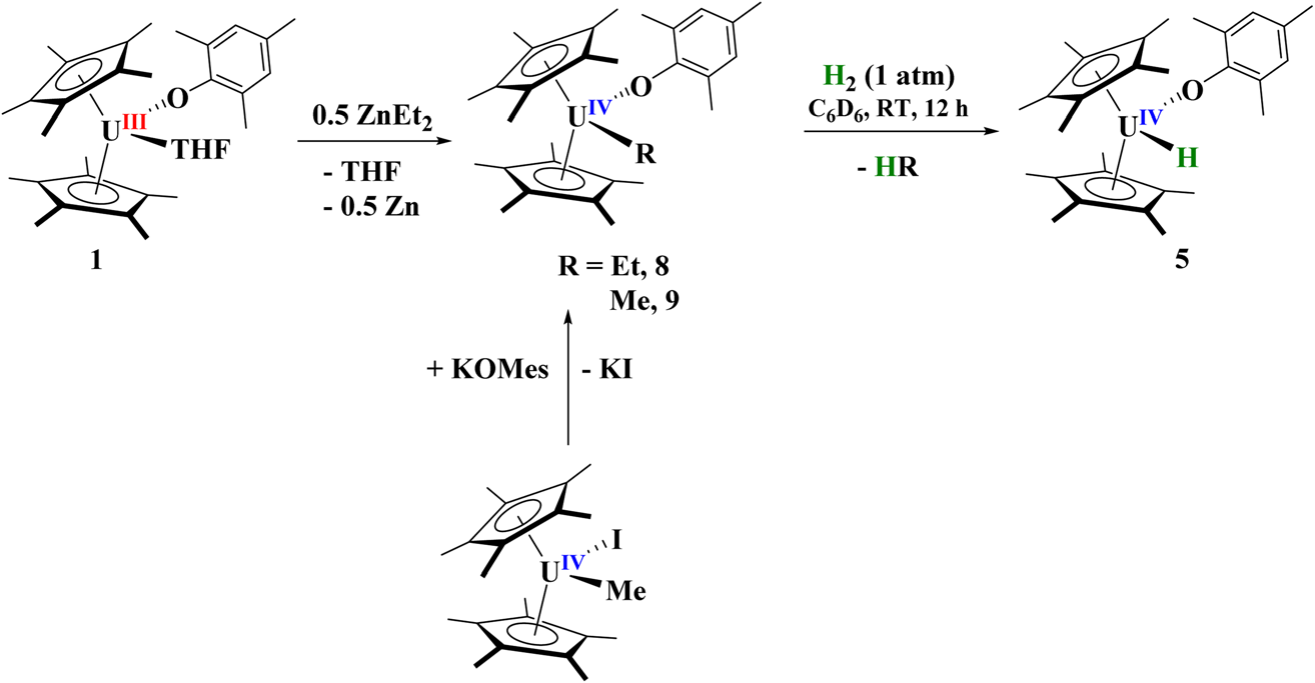
Synthesis of terminal alkyl, [(C_5_Me_5_)_2_(MesO)U(R)], R = Et, 8; Me, 9, and hydride, [(C_5_Me_5_)_2_(MesO)U(H)], 5, complexes.

Next, we examined the reactivity of 2, Scheme 1, and 3, Scheme 2, with dihydrogen. Treatment of 2 with 1 atm H_2_ at 60 °C results in the formation of a red coloured solution. Crystals suitable for X-ray crystallography were grown to reveal a trihydrido-bridged complex, [(C_5_Me_5_)_2_(MesO)U(μ_2_-(H)_3_)–Al(C_5_Me_5_)], 4, Fig. 3. Complex 4 is the result of a formal three-electron metal-based reduction of 1.5 equivalents of H_2_ with two electrons from Al(i) oxidation to Al(iii) and one electron from U(iii) oxidizing to U(iv). An important note is that [(C_5_Me_5_)Al]_4_ does not react with H_2_ alone,^83,84^ thus the formation of 4 is *via* cooperation between the U(iii) and Al(i) metal centres.

The structure of 4 is a rare example of an actinide hydroaluminate complex.^85–88^ It is noteworthy that the hydrides were not modelled but located in the Fourier map and refined. The U–H1, U–H2, and U–H3 distances of 2.33(3), 2.22(3), and 2.25(3) Å, respectively, are consistent with other bridging uranium hydride complexes.^89^ Complex 4 has Al–H bond distances of 1.57(3), 1.54(3), and 1.59(3) Å. The U–O distance of 2.1519(16) Å is similar to other U(iv)-aryloxide complexes previously reported. The U–Al distance shortened from 3.201(3) Å or 3.232(2) Å in 2 to 2.907 Å in 4, but no NBOs were observed between U and Al. Three NBOs between U and each H with approximately 30% U (10–30% 7s with the rest evenly split between 6d and 5f) and 70% hydrogen bonding character were found.

To explore the mechanism in the formation of 4 we investigated the reaction of 1 with H_2_. This reaction is slow at room temperature, but goes to completion after 12 hours at 60 °C. The ^1^H NMR spectrum of the reaction mixture showed two products with one resonance at 272.8 ppm, similar to other terminal U(iv) hydrides previously described by Marks and co-workers.^90^ Thus, we postulated the complex to be [(C_5_Me_5_)_2_(MesO)U(H)], 5. Indeed, when crystals suitable for X-ray diffraction analysis were grown from a saturated Me_3_SiOSiMe_3_ solution at −45 °C, the unit cell matched that of the Zr analogue containing a terminal hydride.^91^ This result is surprising as there is little known about the reduction of H_2_ with the actinides, and nearly all known hydride complexes are formed *via* hydrogenolysis, protonolysis, or the result of Si–H bond activation.^92^ To our knowledge, besides reduction of H_2_ with uranium metal to form UH_3_,^93^ the only molecular examples of H_2_ reduction are a U(ii) complex, [K(2.2.2-cryptand)][(Me_3_SiC_5_H_4_)_3_U] has been reported to reduce H_2_ to form the U(iii) hydride complex, [K(2.2.2-cryptand)][(Me_3_SiC_5_H_4_)_3_U(H)].^94^ Likewise, the Th(ii) complex, [K(18-crown-6)][{(SiMe_3_)_2_C_5_H_3_}_3_Th] with H_2_ results in a mixed-valent thorium(iii/iv) hydride complex.^95^ Hence, a highly reducing divalent actinide centre has been shown in the past to reduce dihydrogen. Even more surprising is that a stirring solution of [(C_5_Me_5_)_2_U(H)_2_] in toluene releases one equivalent of hydrogen with concomitant metal-based reduction to form U(iii).^96–98^

For comparison, reaction of the U–Ga complex, 3, with H_2_ was conducted to afford a red coloured solution with black precipitate. From the ^1^H NMR spectrum, the only uranium-containing product based on paramagnetically shifted resonances were those consistent with 5, Scheme 2. In addition, resonances for C_5_Me_5_H were also observed, thus we conclude that the precipitate was Ga metal. This is the consequence of a heterolytic cleavage of H_2_ with the hydride forming with the electropositive uranium(iv) centre, while the proton is delivered to the (C_5_Me_5_)^1−^ anion. This difference in reactivity is due to the redox potential of monovalent Al and Ga: Al(i) being more favourable to oxidize to Al(iii), while Ga(i) is more easily reduced to Ga(0) in the presence of U(iii) as well as thermal instability of gallium hydrides. This is not surprising given the propensity for Ga(i) to disproportionate when undergoing reaction chemistry.^99,100^ However, in either case, the group 13 metal cooperates with uranium to form a U(iv) hydride.

With the knowledge that 1 can react with H_2_ to form 5, we treated 5 with a species that might feasibly be generated from 2 and H_2_, the trimeric [(C_5_Me_5_)Al(H)_2_]_3_,^101^ which yielded 4 quantitatively, Scheme 1. As mentioned, a second product is observed in the ^1^H NMR spectrum of the reaction of 1 with H_2_, with approximately the same ratio. Four resonances in a 2 : 2 : 2 : 2 pattern, characteristic of a THF ring-opened *n*-butanolate complex, [(C_5_Me_5_)_2_(MesO)U(OCH_2_CH_2_CH_2_CH_3_)], 6, were found and we explored the possibility of the byproduct involving the coordinated THF in 1. Complex 6 can be independently synthesized by oxidizing 1 with half an equivalent of I_2_ to form [(C_5_Me_5_)_2_(MesO)U(i)], 7, followed by salt metathesis with KO^*n*^Bu. The ^1^H NMR spectrum of this product showed identical resonances to those found in the byproduct of 1 with H_2_. Hence, the reduction of H_2_ by 1 forms [(C_5_Me_5_)_2_(MesO)U(X)], X = H, 5, and O^*n*^Bu, 6. We note a recent report by the Anwander group of a lanthanide aluminate complex showing ring-opening of THF.^102^ Complexes 6 and 7 were also structurally characterized by X-ray crystallographic analysis, Fig. S19.†

To examine the kinetics of the reaction of 1 with H_2_, a valved NMR tube charged with 1 atm H_2_ or D_2_, a solution [(C_5_Me_5_)_2_(MesO)U(THF)] in benzene-*d*_6_, was heated to 60 °C. The reaction was observed by taking the ^1^H NMR spectrum every 30 minutes for 5 hours. The disappearance of [(C_5_Me_5_)_2_(MesO)U(THF)] proceeded with first-order or pseudo-first order behaviour. Lowering the pressure of dihydrogen from 0.5 atm and then 0.25 atm did not affect the value of *k*_obs_ for the reaction. The reaction showed a minimal kinetic isotope effect, 1.07(1) *k*_H_2__/*k*_D_2__, and addition of THF (50 : 50 mixture with benzene-*d*_6_) did not change the *k*_obs_. These observations suggest the rate limiting step is possibly an internal rearrangement of [(C_5_Me_5_)_2_(MesO)U(THF)], most likely through a ‘tuck-in’ intermediate,^103–106^ which have been proposed as intermediates in C–H bond activation as well as structurally isolated in thorium^107^ and uranium chemistry.^108–111^ Hence, rather than a reduction of H_2_, this is most likely a hydrogenation-type reaction. While it does not fit our kinetic data, we cannot rule out the possibility of a bimetallic reaction similar to that observed with Zr.^112^

### Synthesis of U(iv) hydride

**Fig. 4:**
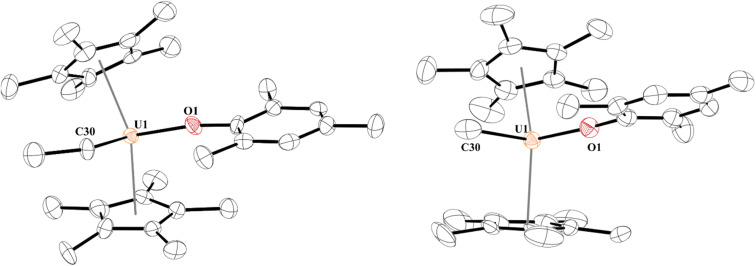
Thermal ellipsoid plot of 8 (left) and 9 (right) shown at the 50% probability level. The hydrogen atoms have been omitted for clarity.

Next, we turned our attention to the U(iv) hydride, 5, since, while terminal hydrides are known,^111,113–117^ they are not well characterized. A higher yielding synthesis of the hydride was desired since the hydride is formed in addition to other byproducts in the reaction of 2 and 3 with H_2_. Reaction of 1 with half an equivalent of ZnEt_2_ leads to the U(iv) ethyl complex, [(C_5_Me_5_)_2_(MesO)U(CH_2_CH_3_)], 8, Scheme 3, in quantitative yield by ^1^H NMR spectroscopy (94% crystalline yield) and can be readily separated from Zn metal. We also isolated the methyl complex, [(C_5_Me_5_)_2_(MesO)U(CH_3_)], 9, by treatment of [(C_5_Me_5_)_2_U(CH_3_)(I)]^118^ with KOMes. The ^1^H NMR spectrum of each complex are similar with a (C_5_Me_5_)^1−^ resonance at 4.31 ppm and 4.41 ppm for 8 and 9, respectively, and a characteristic peak at −182.76 ppm for the methylene of the ethyl group and −183.1 ppm for the methyl. Crystals suitable for X-ray crystallographic analysis were grown from a saturated pentane solution at −45 °C. While terminal ethyl complexes of the f elements have been reported,^103,104,119,120^ this is the first solid-state structure determination of an ethyl complex^121–123^ with an actinide and a rare example of an f element complex containing a β-hydrogen. We do not observe any β-hydride elimination with 8, and it is stable at room temperature. Complexes 8 and 9 show the typical pseudo-tetrahedral geometry (Fig. 4). The U–C(ethyl) bond distance of 2.42(1) Å and U–C(methyl) bond length of 2.474(3) Å are similar to other U–C(alkyl) complexes such as 2.424(7) and 2.414(7) Å in (C_5_Me_5_)_2_U(CH_3_)_2_.^124^

Hydrogenation of 8 or 9 leads to the clean formation of 5 in high yield (Scheme 3). While 5 was shown to be isostructural with the zirconium analogue having a terminal hydride, we wanted to investigate the hydride more closely. When 8 or 9 are reacted with D_2_, a resonance in the ^2^H NMR spectrum at 264.6 ppm is observed, similar to the 272.8 ppm observed in the ^1^H NMR spectrum. However, we do see several resonances in the ^2^H NMR spectrum, indicating that scrambling is occurring into the (C_5_Me_5_)^1−^ and aryloxide ligands. This is well known to occur in (C_5_Me_5_)^1−^ ligated systems^96,108^ and it is thought to occur through a ‘tuck-in’ intermediate,^103,105^ thus providing further evidence for our ‘tuck-in’ mechanism in the reaction of [(C_5_Me_5_)_2_(MesO)U(THF)] with H_2_, which showed first-order kinetics. We see no evidence of an additional species in the ^1^H or ^2^H NMR spectra, which we would expect if a monomer–dimer equilibrium was occurring which is the case for [(C_5_Me_5_)_2_U(H)]_2_ and [(C_5_Me_5_)_2_U(H)].^96–98^ In the IR spectrum, an absorption at 1384 cm^−1^ is observed which we assign to the U–H stretch, which is similar to other literature values as well as that absorption being absent in the deuteride spectrum.^89^ An absorption with medium intensity at 1017 cm^−1^ is observed in the IR spectrum which is close to the ∼1000 cm^−1^ that is anticipated based on the reduced mass, and we therefore assign this to the U–D stretch. These U–H and U–D stretching frequencies are similar to the 1395 cm^−1^ and 1015 cm^−1^, respectively, reported in [(C_5_H_4_^*t*^Bu)_3_U(H)].^116^ When 5 is treated with CCl_4_, the formation of CHCl_3_ is observed in the ^1^H NMR spectrum. If stored in a glove box at −25 °C, complex 5 is stable for weeks. This evidence establishes complex 5 to be a thermally and kinetically stable, U(iv) terminal hydride complex.

## Conclusions

The synthesis of uranium–metal bonded complexes, [(C_5_Me_5_)_2_(MesO)UM(C_5_Me_5_)], M = Al, Ga, has been reported. When treated with H_2_, the U–Al compound forms a trihydroaluminate complex along with a U(iv) hydride. In the case of the U–Ga complex, Ga(i) is reduced to Ga metal while the hydride migrates to make the same U(iv) hydride previously observed with the U–Al complex, and the proton from H_2_ forms C_5_Me_5_H. These are rare examples of dihydrogen activation with a uranium complex as well as observing cooperative chemistry between a metal and an f element. We further investigated the formation of the U(iv) hydride through kinetic and isotopic labelling studies, which indicate that the reaction of 1 with H_2_ is not a reduction of H_2_, but most likely the result of a hydrogenation reaction of a U(iv) tuck-in complex. Further, we found a higher yielding synthesis of the hydride through hydrogenation of a rare ethyl complex, formed from the alkylation with ZnEt_2_. We continue to search for new examples of cooperative chemistry with our uranium–metal bonded systems, and hope this report inspires further dihydrogen-based activation in f element chemistry.

## Data availability

The associated ESI† file contains the data supporting this article (synthetic procedures, characterization data, NMR spectra, and structure determination).

## Author contributions

R. J. Ward and P. Rungthanaphatsophon performed the synthetic experimental work; P. Huang performed and interpreted the computational studies; S. P. Kelley recorded and interpreted the X-ray diffraction analysis; J. R. Walensky conceptualized the research, acquired funding, and supervised the work; all authors revised and edited the manuscript. All authors have read and agreed to the published version of the manuscript.

## Conflicts of interest

There are no conflicts to declare.

## Supplementary Material

SC-014-D3SC04857H-s001

SC-014-D3SC04857H-s002

SC-014-D3SC04857H-s003

SC-014-D3SC04857H-s004

SC-014-D3SC04857H-s005

## References

[cit1] Khusnutdinova J. R., Milstein D. (2015). Angew. Chem., Int. Ed..

[cit2] Wheatley N., Kalck P. (1999). Chem. Rev..

[cit3] Gade L. H. (2000). Angew. Chem., Int. Ed..

[cit4] Cooper B. G., Napoline J. W., Thomas C. M. (2012). Catal. Rev..

[cit5] Thomas C. M. (2011). Comments Inorg. Chem..

[cit6] Wang Q., Brooks S. H., Liu T., Tomson N. C. (2021). Chem. Commun..

[cit7] Fernández S., Fernando S., Planas O. (2023). Dalton Trans..

[cit8] Krogman J. P., Foxman B. M., Thomas C. M. (2011). J. Am. Chem. Soc..

[cit9] Wu B., Bezpalko M. W., Foxman B. M., Thomas C. M. (2015). Chem. Sci..

[cit10] Zhang H., Hatzis G. P., Moore C. E., Dickie D. A., Bezpalko M. W., Foxman B. M., Thomas C. M. (2019). J. Am. Chem. Soc..

[cit11] Gade L. H., Memmler H., Kauper U., Schneider A., Fabre S., Bezougli I., Lutz M., Galka C., Scowen I. J., McPartlin M. (2000). Chem. – Eur. J..

[cit12] Baranger A. M., Hanna T. A., Bergman R. G. (1995). J. Am. Chem. Soc..

[cit13] Mazzacano T. J., Mankad N. P. (2013). J. Am. Chem. Soc..

[cit14] Minasian S. G., Krinsky J. L., Williams V. A., Arnold J. (2008). J. Am. Chem. Soc..

[cit15] Minasian S. G., Krinsky J. L., Rinehart J. D., Copping R., Tyliszczak T., Janousch M., Shuh D. K., Arnold J. (2009). J. Am. Chem. Soc..

[cit16] Winston M. S., Batista E. R., Yang P., Tondreau A. M., Boncella J. M. (2016). Inorg. Chem..

[cit17] Rookes T. M., Wildman E. P., Balázs G., Gardner B. M., Wooles A. J., Gregson M., Tuna F., Scheer M., Liddle S. T. (2018). Angew. Chem., Int. Ed..

[cit18] Tarlton M. L., Fajen O. J., Kelley S. P., Kerridge A., Malcomson T., Morrison T. L., Shores M. P., Xhani X., Walensky J. R. (2021). Inorg. Chem..

[cit19] Ayres A. J., Zegke M., Ostrowski J. P. A., Tuna F., McInnes E. J. L., Wooles A. J., Liddle S. T. (2018). Chem. Commun..

[cit20] Gardner B. M., McMaster J., Moro F., Lewis W., Blake A. J., Liddle S. T. (2011). Chem. – Eur. J..

[cit21] Fortier S., Aguilar-Calderón J. R., Vlaisavljevich B., Metta-Magaña A. J., Goos A. G., Botez C. E. (2017). Organometallics.

[cit22] Bucaille A., Le Borgne T., Ephritikhine M., Daran J.-C. (2000). Organometallics.

[cit23] Monreal M. J., Khan S. I., Kiplinger J. L., Diaconescu P. L. (2011). Chem. Commun..

[cit24] Gardner B. M., Patel D., Cornish A. D., McMaster J., Lewis W., Blake A. J., Liddle S. T. (2011). Chem. – Eur. J..

[cit25] Patel D., Moro F., McMaster J., Lewis W., Blake A. J., Liddle S. T. (2011). Angew. Chem., Int. Ed..

[cit26] Napoline J. W., Kraft S. J., Matson E. M., Fanwick P. E., Bart S. C., Thomas C. M. (2013). Inorg. Chem..

[cit27] Ward A. L., Lukens W. W., Lu C. C., Arnold J. (2014). J. Am. Chem. Soc..

[cit28] Hlina J. A., Wells J. A. L., Pankhurst J. R., Love J. B., Arnold P. L. (2017). Dalton Trans..

[cit29] Hlina J. A., Pankhurst J. R., Kaltsoyannis N., Arnold P. L. (2016). J. Am. Chem. Soc..

[cit30] Fortier S., Walensky J. R., Wu G., Hayton T. W. (2011). J. Am. Chem. Soc..

[cit31] Fang W., Douair I., Hauser A., Li K., Zhao Y., Roesky P. W., Wang S., Maron L., Zhu C. (2022). CCS Chem..

[cit32] Sternal R. S., Brock C. P., Marks T. J. (1985). J. Am. Chem. Soc..

[cit33] Ritchey J. M., Zozulin A. J., Wrobleski D. A., Ryan R. R., Wasserman H. J., Moody D. C., Paine R. T. (1985). J. Am. Chem. Soc..

[cit34] Bianconi P. A., Williams I. D., Engeler M. P., Lippard S. J. (1986). J. Am. Chem. Soc..

[cit35] Yang P., Zhou E., Hou G., Zi G., Ding W., Walter M. D. (2016). Chem. – Eur. J..

[cit36] Tarlton M. L., Kelley S. P., Walensky J. R. (2021). Acta Crystallogr., Sect. E: Crystallogr. Commun..

[cit37] Feng G., Zhang M., Shao D., Wang X., Wang S., Maron L., Zhu C. (2019). Nat. Chem..

[cit38] Feng G., Zhang M., Wang P., Wang S., Maron L., Zhu C. (2019). Proc. Natl. Acad. Sci. U. S. A..

[cit39] Feng G., McCabe K. N., Wang S., Maron L., Zhu C. (2020). Chem. Sci..

[cit40] Xin X., Douair I., Zhao Y., Wang S., Maron L., Zhu C. (2020). J. Am. Chem. Soc..

[cit41] Ostrowski J. P. A., Wooles A. J., Liddle S. T. (2021). Inorganics.

[cit42] Liddle S. T., Mills D. P. (2009). Dalton Trans..

[cit43] Liddle S. T., McMaster J., Mills D. P., Blake A. J., Jones C., Woodul W. D. (2009). Angew. Chem., Int. Ed..

[cit44] Porchia M., Casellato U., Ossola F., Rossetto G., Zanella P., Graziani R. (1986). J. Chem. Soc., Chem. Commun..

[cit45] Gardner B. M., McMaster J., Lewis W., Liddle S. T. (2009). Chem. Commun..

[cit46] Shima T., Luo Y., Stewart T., Bau R., McIntyre G. J., Mason S. A., Hou Z. (2011). Nat. Chem..

[cit47] Zhu Q., Fang W., Maron L., Zhu C. (2022). Acc. Chem. Res..

[cit48] Diaconescu P. L. (2010). Acc. Chem. Res..

[cit49] Odom A. L., Arnold P. L., Cummins C. C. (1998). J. Am. Chem. Soc..

[cit50] Xin X., Douair I., Rajeshkumar T., Zhao Y., Wang S., Maron L., Zhu C. (2022). Nat. Commun..

[cit51] Fang X., Li X., Hou Z., Assoud J., Zhao R. (2009). Organometallics.

[cit52] Yuan Y., Chen Y., Li G., Xia W. (2010). Organometallics.

[cit53] Wang X., Leng X., Chen Y. (2015). Dalton Trans..

[cit54] Meng Y.-S., Wang C.-H., Zhang Y.-Q., Leng X.-B., Wang B.-W., Chen Y.-F., Gao S. (2016). Inorg. Chem. Front..

[cit55] Cui P., Chen Y. (2016). Coord. Chem. Rev..

[cit56] Barisic D., Schneider D., Maichle-Mössmer C., Anwander R. (2019). Angew. Chem., Int. Ed..

[cit57] Gamer M. T., Roesky P. W., Konchenko S. N., Nava P., Ahlrichs R. (2006). Angew. Chem., Int. Ed..

[cit58] Wiecko M., Roesky P. W. (2007). Organometallics.

[cit59] Sanden T., Gamer M. T., Fagin A. A., Chudakova V. A., Konchenko S. N., Fedushkin I. L., Roesky P. W. (2012). Organometallics.

[cit60] Arnold P. L., Liddle S. T., McMaster J., Jones C., Mills D. P. (2007). J. Am. Chem. Soc..

[cit61] Liddle S. T., Mills D. P., Gardner B. M., McMaster J., Jones C., Woodul W. D. (2009). Inorg. Chem..

[cit62] Saleh L. M. A., Birjkumar K. H., Protchenko A. V., Schwarz A. D., Aldridge S., Jones C., Kaltsoyannis N., Mountford P. (2011). J. Am. Chem. Soc..

[cit63] Sugita K., Yamashita M. (2020). Chem. – Eur. J..

[cit64] Paprocki V., Hrobárik P., Harriman K. L. M., Luff M. S., Kupfer T., Kaupp M., Murugesu M., Braunschweig H. (2020). Angew. Chem., Int. Ed..

[cit65] Ward R. J., Del Rosal I., Chirdon D. N., Kelley S. P., Tarlton M. L., Maron L., Walensky J. R. (2020). Inorg. Chem..

[cit66] Ward R. J., Pividori D., Carpentier A., Tarlton M. L., Kelley S. P., Maron L., Meyer K., Walensky J. R. (2021). Organometallics.

[cit67] Ward R. J., Kelley S. P., Lukens W. W., Walensky J. R. (2022). Organometallics.

[cit68] Ward R. J., Rosal I. d., Kelley S. P., Maron L., Walensky J. R. (2023). Chem. Sci..

[cit69] Poitras A. M., Knight S. E., Bezpalko M. W., Foxman B. M., Thomas C. M. (2018). Angew. Chem., Int. Ed..

[cit70] Navarro M. and Campos J., in Advances in Organometallic Chemistry, ed. P. J. Pérez, Academic Press, 2021, vol. 75, pp. 95–148

[cit71] Thomas C. M., Napoline J. W., Rowe G. T., Foxman B. M. (2010). Chem. Commun..

[cit72] Ramirez B. L., Sharma P., Eisenhart R. J., Gagliardi L., Lu C. C. (2019). Chem. Sci..

[cit73] Devillard M., Declercq R., Nicolas E., Ehlers A. W., Backs J., Saffon-Merceron N., Bouhadir G., Slootweg J. C., Uhl W., Bourissou D. (2016). J. Am. Chem. Soc..

[cit74] Hidalgo N., Moreno J. J., Pérez-Jiménez M., Maya C., López-Serrano J., Campos J. (2020). Chem. – Eur. J..

[cit75] Evans M. J., Anker M. D., McMullin C. L., Neale S. E., Coles M. P. (2021). Angew. Chem., Int. Ed..

[cit76] Hidalgo N., de la Cruz-Martínez F., Martín M. T., Nicasio M. C., Campos J. (2022). Chem. Commun..

[cit77] Liu H.-Y., Neale S. E., Hill M. S., Mahon M. F., McMullin C. L., Morrison B. L. (2023). Chem. Commun..

[cit78] Wedal J. C., Ziller J. W., Furche F., Evans W. J. (2022). Inorg. Chem..

[cit79] Cordero B., Gómez V., Platero-Prats A. E., Revés M., Echeverría J., Cremades E., Barragán F., Alvarez S. (2008). Dalton Trans..

[cit80] Van der Sluys W. G., Sattelberger A. P. (1990). Chem. Rev..

[cit81] Avens L. R., Burns C. J., Butcher R. J., Clark D. L., Gordon J. C., Schake A. R., Scott B. L., Watkin J. G., Zwick B. D. (2000). Organometallics.

[cit82] Evans W. J., Montalvo E., Ziller J. W., DiPasquale A. G., Rheingold A. L. (2010). Inorg. Chem..

[cit83] Himmel H.-J., Vollet J. (2002). Organometallics.

[cit84] Chu T., Korobkov I., Nikonov G. I. (2014). J. Am. Chem. Soc..

[cit85] Annby U., Gronowitz S., Hallberg A. (1989). J. Organomet. Chem..

[cit86] Le Marechal J.-F., Ephritikhine M., Folcher G. (1986). J. Organomet. Chem..

[cit87] Ossola F., Brianese N., Porchia M., Rossetto G., Zanella P. (1986). J. Organomet. Chem..

[cit88] Ossola F., Brianese N., Porchia M., Rossetto G., Zanella P. (1990). J. Chem. Soc., Dalton Trans..

[cit89] Ephritikhine M. (1997). Chem. Rev..

[cit90] Lin Z., Marks T. J. (1990). J. Am. Chem. Soc..

[cit91] Jian Z., Kehr G., Daniliuc C. G., Wibbeling B., Wiegand T., Siedow M., Eckert H., Bursch M., Grimme S., Erker G. (2017). J. Am. Chem. Soc..

[cit92] Ren W., Zhou E., Fang B., Zi G., Fang D.-C., Walter M. D. (2014). Chem. Sci..

[cit93] Burke J. E., Smith C. S. (1947). J. Am. Chem. Soc..

[cit94] MacDonald M. R., Fieser M. E., Bates J. E., Ziller J. W., Furche F., Evans W. J. (2013). J. Am. Chem. Soc..

[cit95] Langeslay R. R., Fieser M. E., Ziller J. W., Furche F., Evans W. J. (2016). J. Am. Chem. Soc..

[cit96] Fagan P. J., Manriquez J. M., Maatta E. A., Seyam A. M., Marks T. J. (1981). J. Am. Chem. Soc..

[cit97] Evans W. J., Miller K. A., Kozimor S. A., Ziller J. W., DiPasquale A. G., Rheingold A. L. (2007). Organometallics.

[cit98] Pagano J. K., Dorhout J. M., Czerwinski K. R., Morris D. E., Scott B. L., Waterman R., Kiplinger J. L. (2016). Organometallics.

[cit99] Jurca T., Dawson K., Mallov I., Burchell T., Yap G. P. A., Richeson D. S. (2010). Dalton Trans..

[cit100] Baker R. J., Jones C. (2005). Dalton Trans..

[cit101] Ganesamoorthy C., Loerke S., Gemel C., Jerabek P., Winter M., Frenking G., Fischer R. A. (2013). Chem. Commun..

[cit102] Bonath M., Birkelbach V. M., Stuhl C., Maichle-Mössmer C., Anwander R. (2021). Chem. Commun..

[cit103] Thompson M. E., Baxter S. M., Bulls A. R., Burger B. J., Nolan M. C., Santarsiero B. D., Schaefer W. P., Bercaw J. E. (1987). J. Am. Chem. Soc..

[cit104] Watson P. L. (1983). J. Am. Chem. Soc..

[cit105] Watson P. L., Parshall G. W. (1985). Acc. Chem. Res..

[cit106] Bruno J. W., Smith G. M., Marks T. J., Fair C. K., Schultz A. J., Williams J. M. (1986). J. Am. Chem. Soc..

[cit107] Evans W. J., Walensky J. R., Ziller J. W. (2009). Chem. – Eur. J..

[cit108] Evans W. J., Miller K. A., DiPasquale A. G., Rheingold A. L., Stewart T. J., Bau R. (2008). Angew. Chem., Int. Ed..

[cit109] Montalvo E., Ziller J. W., DiPasquale A. G., Rheingold A. L., Evans W. J. (2010). Organometallics.

[cit110] Takase M. K., Siladke N. A., Ziller J. W., Evans W. J. (2011). Organometallics.

[cit111] Higgins J. A., Cloke F. G. N., Roe S. M. (2013). Organometallics.

[cit112] Bradley C. A., Veiros L. F., Pun D., Lobkovsky E., Keresztes I., Chirik P. J. (2006). J. Am. Chem. Soc..

[cit113] Turner H. W., Andersen R. A., Zalkin A., Templeton D. H. (1979). Inorg. Chem..

[cit114] Simpson S. J., Turner H. W., Andersen R. A. (1981). Inorg. Chem..

[cit115] Berthet J.-C., Le Maréchal J.-F., Ephritikhine M. (1991). J. Chem. Soc., Chem. Commun..

[cit116] Berthet J.-C., Le Maréchal J.-F., Lance M., Nierlich M., Vigner J., Ephritikhine M. (1992). J. Chem. Soc., Dalton Trans..

[cit117] Turner H. W., Simpson S. J., Andersen R. A. (1979). J. Am. Chem. Soc..

[cit118] Rungthanaphatsophon P., Huang P., Walensky J. R. (2018). Organometallics.

[cit119] Schumann H., Genthe W., Bruncks N. (1981). Angew. Chem., Int. Ed..

[cit120] Evans W. J., Meadows J. H., Hunter W. E., Atwood J. L. (1984). J. Am. Chem. Soc..

[cit121] Hayes P. G., Piers W. E., Lee L. W. M., Knight L. K., Parvez M., Elsegood M. R. J., Clegg W. (2001). Organometallics.

[cit122] Fryzuk M. D., Giesbrecht G., Rettig S. J. (1996). Organometallics.

[cit123] Gountchev T. I., Tilley T. D. (1999). Organometallics.

[cit124] Jantunen K. C., Burns C. J., Castro-Rodriguez I., Da Re R. E., Golden J. T., Morris D. E., Scott B. L., Taw F. L., Kiplinger J. L. (2004). Organometallics.

