# Supplementary material for: Cooperative dihydrogen activation with unsupported uranium–metal bonds and characterization of a terminal U(iv) hydride
Source: Chem Sci. 2023 Oct 16;14(43):12255–63. doi: 10.1039/d3sc04857h (PMC10631237; doi:10.1039/d3sc04857h)
Supplement: SC-014-D3SC04857H-s001 [file SC-014-D3SC04857H-s001.pdf]

Electronic Supplementary Material (ESI) for Chemical Science.
This journal is © The Royal Society of Chemistry 2023

Supporting Information for

Cooperative Dihydrogen Activation with Unsupported Uranium-Metal Bonds and Characterization of a Terminal U(IV) Hydride

Robert J. Ward,[a] Pokpong Rungthanaphatsophon,[a] Patrick Huang,[b] Steven P. Kelley,[a] and Justin R. Walensky[a]

[a] Department of Chemistry, University of Missouri, Columbia, MO 65211 USA

[b] Department of Chemistry and Biochemistry, California State University, East Bay, Hayward, CA 94542 USA

Table of Contents

General considerations……………………………………………………………………………S2
Synthesis of [$(C_5Me_5)_2$(MesO)UAl(C_5Me_5)], **2**……………………………………………………...S2
Figure S1: FTIR vibrational spectrum of [$(C_5Me_5)_2$(MesO)UAl(C_5Me_5)], **2**..S2
Figure S2: ^{1}H NMR spectrum of [$(C_5Me_5)_2$(MesO)UAl(C_5Me_5)] in toluene-d_8. ....S3
Figure S3: FTIR vibrational spectrum of [$(C_5Me_5)_2$(MesO)UGa(C_5Me_5)]………………………………S4
Figure S4: NMR spectrum of [$(C_5Me_5)_2$(MesO)UGa(C_5Me_5)] in benzene-d_6.
Figure S5: Plot of the ^{1}H NMR chemical shift (ppm) versus temperature (K) of [$(C_5Me_5)_2$(MesO)UGa(C_5Me_5)], **3**, with [(C_5Me_5)Ga] fragment in blue and [$(C_5Me_5)_2$U] fragment in orange……………………………………………………………………………………………...S5
Figure S6: Plot of ^{1}H NMR chemical shift (ppm) versus temperature (K) for the $(C_5Me_5)_2$U protons in the [$(C_5Me_5)_2$(MesO)UGa(C_5Me_5)], **3**, system with n equivalents of [(C_5Me_5)Ga], in d_8-toluene………..…S6

Figure S7. Variable temperature ^{1}H NMR spectrum of reaction of **1** with [(C_5Me_5)Ga]…………………S6

Figure S8: ^{1}H NMR spectrum of [$(C_5Me_5)_2$(MesO)U{μ-H_3Al(C_5Me_5)}] in benzene-d_6…………………S7
Figure S9: FTIR vibrational spectrum of [$(C_5Me_5)_2$(MesO)U{μ-H_3Al(C_5Me_5)}]…………………..….S8

Figure S10: ^{1}H NMR spectra of [$(C_5Me_5)_2$(MesO)U(H)] in benzene-d_6..S9
Figure S11: First order kinetics graph for the reaction of [$(C_5Me_5)_2$(MesO)U(THF)] with H_2. ....S9
Figure S12: FTIR vibrational spectrum of [$(C_5Me_5)_2$(MesO)U(H)]. S10
Figure S13: FTIR vibrational spectrum of [$(C_5Me_5)_2$(MesO)U(D)]. S10
Figure S14: ^{1}H NMR spectrum of [$(C_5Me_5)_2$(MesO)U(O-^{n}Bu)], **6**...S11
Figure S15: Zoomed in ^{1}H NMR spectrum of [$(C_5Me_5)_2$(MesO)U(O-^{n}Bu)]. S11
Figure S16: FTIR vibrational spectrum of [$(C_5Me_5)_2$(MesO)U(O-^{n}Bu)]. S12
Figure S17: ^{1}H NMR spectrum of [$(C_5Me_5)_2$(MesO)U(I)] in benzene-d_6. ....S13
Figure S18: FTIR vibrational spectrum of [$(C_5Me_5)_2$(MesO)U(I)]. S13
Figure S19: Thermal ellipsoid of **6** (left) and **7** (right) shown at the 50% probability level. The hydrogen atoms have been omitted for clarity…………………………………………………………………S14

Figure S20: ^{1}H NMR spectrum of [$(C_5Me_5)_2$(MesO)U(CH_2CH_3)]. S14
Figure S21: FTIR vibrational spectrum of [$(C_5Me_5)_2$(MesO)U(CH_2CH_3)]..S15
Figure S22: ^{1}H NMR spectrum of [$(C_5Me_5)_2$(MesO)U(CH_3)] in benzene-d_6. ....S16
Figure S23: FTIR vibrational spectrum of [$(C_5Me_5)_2$(MesO)U(CH_3)]. S17

Figure S24: Detection of $CHCl_3$ (6.18 ppm) from $[(C_5Me_5)_2(MesO)U(H)]$ and CCl_4 in C_6D_6. ....S18
Crystal Structure Refinement Details……………………………………………………………S19

Table S1. X-ray crystallographic details for complexes **2-4** and **6**………………………………...……..S20
Table S2. X-ray crystallographic details for complexes **7-9**…………………………………………S21
Table S3. Selected bond angles (deg) in complexes **2-4**, **6-9**…………………………………………S22
Computational Details………………………………………………………………………S22
References…………………………………………………………………………………S22

General Considerations. All syntheses were carried out under an N_2 atmosphere using glovebox and Schlenk techniques unless otherwise stated. All non-deuterated solvents used were dried by passing through a solvent purification system (MBraun, USA), and stored over sieves, and potassium for aromatic and aliphatic solvents, or calcium hydride for ethereal solvents. $[(C_5Me_5)_2UI(THF)]$,[1] $[(C_5Me_5)_2U(CH_3)(I)]$,[2] $[(C_5Me_5)AlH_2]_3$,[3] $[(C_5Me_5)Al]_4$,[4] $[(C_5Me_5)Ga]$,[5] and $[(C_5Me_5)_2(MesO)U(THF)]$[6] were prepared according to literature methods. Potassium *n*-butoxide was prepared from the stoichiometric reaction of *n*-butanol with $KN(SiMe_3)_2$ in toluene. Benzene-d_6 (Cambridge Isotope Laboratories) were degassed by three freeze−pump−thaw cycles and stored over molecular sieves. Toluene-d_8 (Cambridge Isotope Laboratories) and hexamethyldisiloxane, was distilled from potassium-ketyl. All ^1H spectra were taken on 600 MHz or 300 MHz Bruker spectrometers. All NMR chemical shifts are reported in ppm. ^1H NMR chemical shifts were referenced internally to the residual solvent peaks. IR were taken on a Nicolet Summit Pro FtIR spectrometer with, using a KBr pellet. Elemental analysis was performed at the University of Missouri, Columbia on a Carlo Erba 1108 elemental analyzer, outfitted with an A/D converter for analysis using Eager Xperience software.

Caution! Depleted uranium (primarily U-238) is an α emitting radiometal with a half-life of 4.47×10^9 years. All work was performed in a radiological laboratory with appropriate personal protective and counting equipment.

Synthesis of $[(C_5Me_5)_2(MesO)UAl(C_5Me_5)]$, 2. $[(C_5Me_5)_2(MesO)U(THF)]$ (70.6 mg, 0.0987 mmol) was dissolved in 5 mL of toluene and combined with $[(C_5Me_5)Al]_4$ (19.0 mg, 0.0293 mmol) in 5 mL of toluene. The combined toluene solutions were stirred and heated to about 60 °C, to fully dissolve the $[(C_5Me_5)Al]_4$. Immediately after, volatiles were removed, slowly, under reduced pressure with continuous stirring. The resulting green film can be crystalized from a concentrated solution of pentane at –14 °C, yielding 25.7 mg $[(C_5Me_5)_2(MesO)UAl(C_5Me_5)]$ (0.0319 mmol, 32%). X-ray quality crystals of the toluene solvate can be grown from the crystalline sample redissolved in toluene and layered with hexamethyldisiloxane, and stored at –14 °C. ^1H NMR (C_7D_8, 254 K): δ -27.09 (s, 3H, o-Ar-C**H$_3$**), -14.45 (s, 3H, o-Ar-C**H$_3$**), -12.96 (s, 30H, U(C$_5$(C**H**$_3$)$_5$), -10.45 (s, 15H, Al(C$_5$(C**H**$_3$)$_5$), 4.31 (s, 3H, p-Ar-C**H**$_3$), 7.54 (s, 1H, m-Ar-**H**), 10.79 (s, 1H, m-Ar-**H**). IR (KBr, cm^{-1}): 2933 (s), 2912 (s), 2853 (s), 1654 (m), 1598 (w), 1473 (s), 1385 (m), 1308 (s), 1252 (s), 1159 (m), 1093 (mb), 1022 (m), 823 (s), 705 (m), 514 (w), 456(m). Anal. Calcd. theory (found) for $C_{39}H_{56}AlUO$, C 58.13 (58.35%), H 7.00 (7.02%).

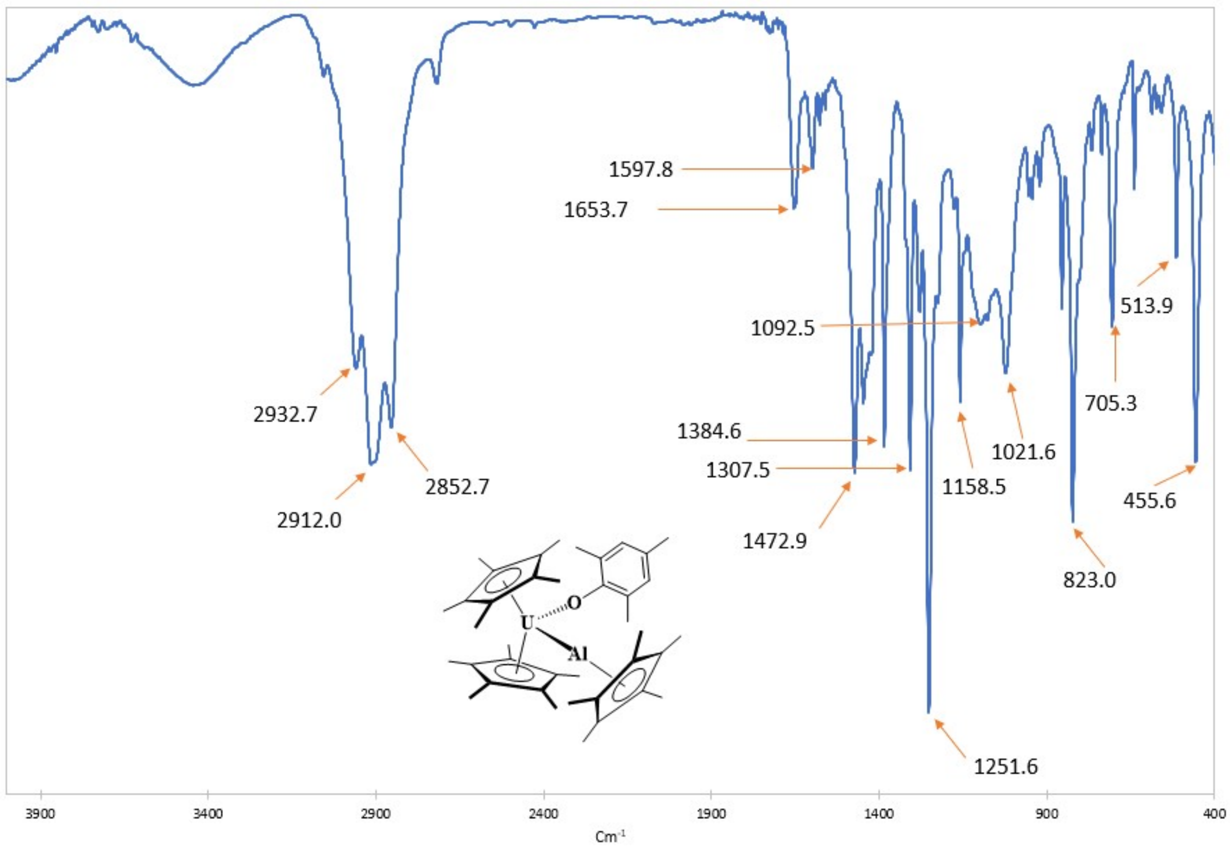

Figure S1: FTIR vibrational spectrum of $[(C_5Me_5)_2(MesO)UAl(C_5Me_5)]$, **2**.

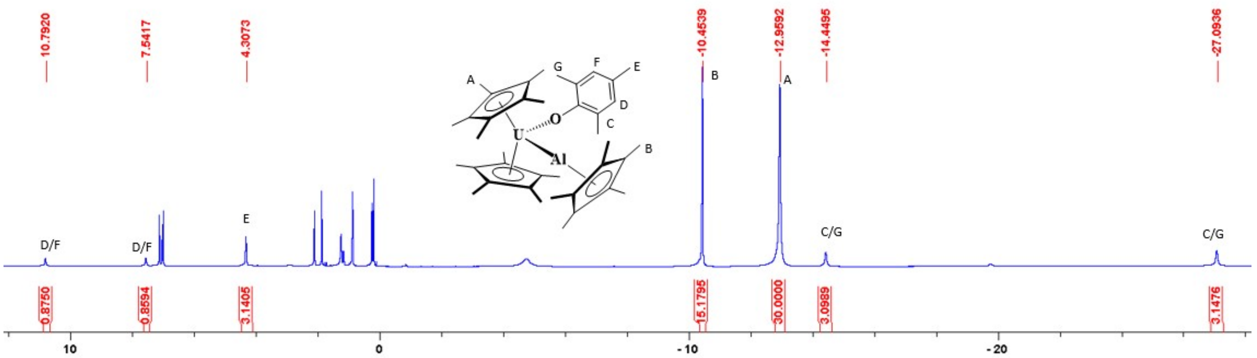

Figure S2: ^{1}H NMR spectrum of $[(C_5Me_5)_2(MesO)UAl(C_5Me_5)]$ in toluene-d_8.

Synthesis of [(C_5Me_5)$_2$(MesO)UGa(C_5Me_5)], 3. [(C_5Me_5)$_2$(MesO)U(THF)] (142.6 mg, 0.1994 mmol) was dissolved in 5 mL of pentane and combined with [(C_5Me_5)Ga] (40.9 mg, 0.1995 mmol) in 5 mL of pentane. The combined toluene solutions were stirred for 20 minutes. Volatiles were removed, under reduced pressure with continuous stirring. The resulting green powder can be crystalized from a concentrated solution of pentane at –14 °C, yielding 69.7 mg [(C_5Me_5)$_2$(MesO)UGa(C_5Me_5)] (0.0821 mmol, 41%). X-ray quality crystals can be grown from pentane at –14 °C. ^{1}H NMR (C_6D_6, 300 K): -18.76 (bs, 6H, o-Ar-C**H**$_3$), -11.02 (bs, 30H, U(C_5(C**H**$_3$)$_5$), -5.64 (s, 15H, Ga(C_5(C**H**$_3$)$_5$), 3.86 (s, 3H, p-Ar-C**H**$_3$), 8.53 (s, 2H, m-Ar-**H**). IR (KBr, cm^{-1}): 2934 (s), 2910 (s), 2854 (s), 1608 (w), 1474 (s), 1432 (m), 1384 (m), 1308 (s), 1246 (s), 1158 (m), 1066 (wb), 1119 (wb), 824 (s), 516 (m). Anal. Calcd. theory (found) for $C_{39}H_{56}$GaUO, C 55.20 (55.06%), H 6.65 (6.47%).

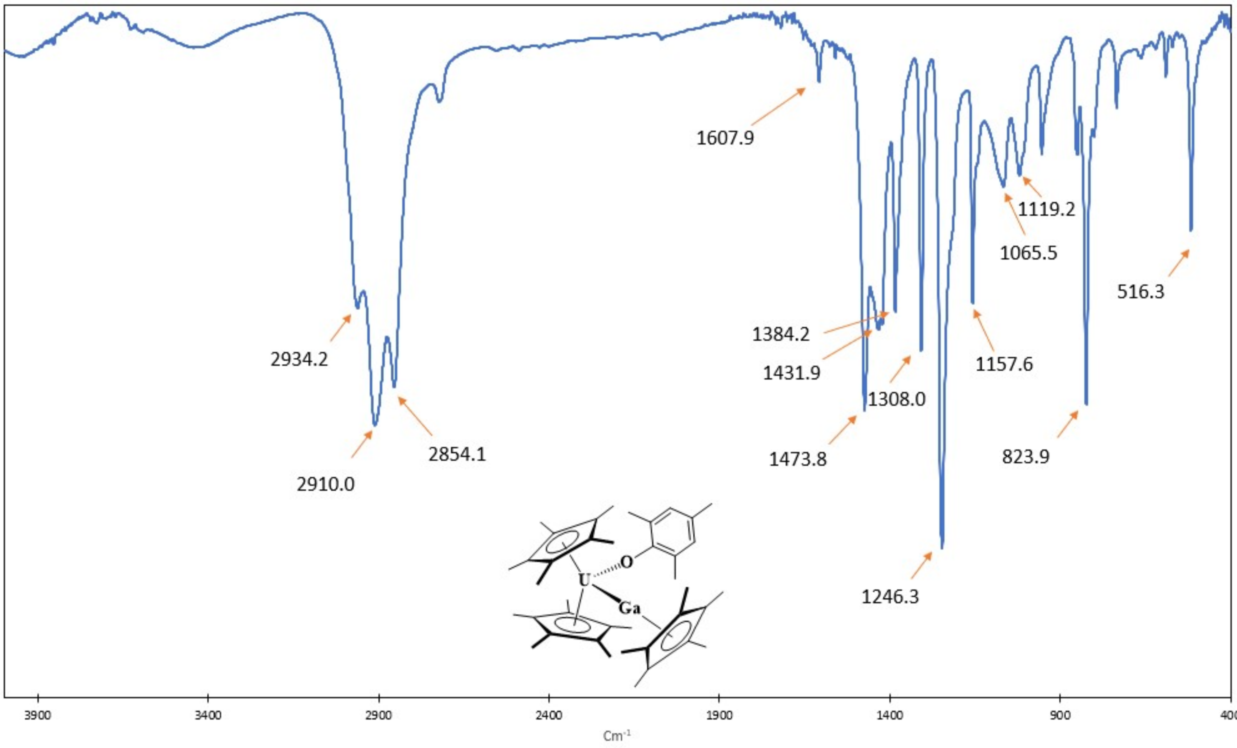

Figure S3: FTIR vibrational spectrum of [(C_5Me_5)$_2$(MesO)UGa(C_5Me_5)].

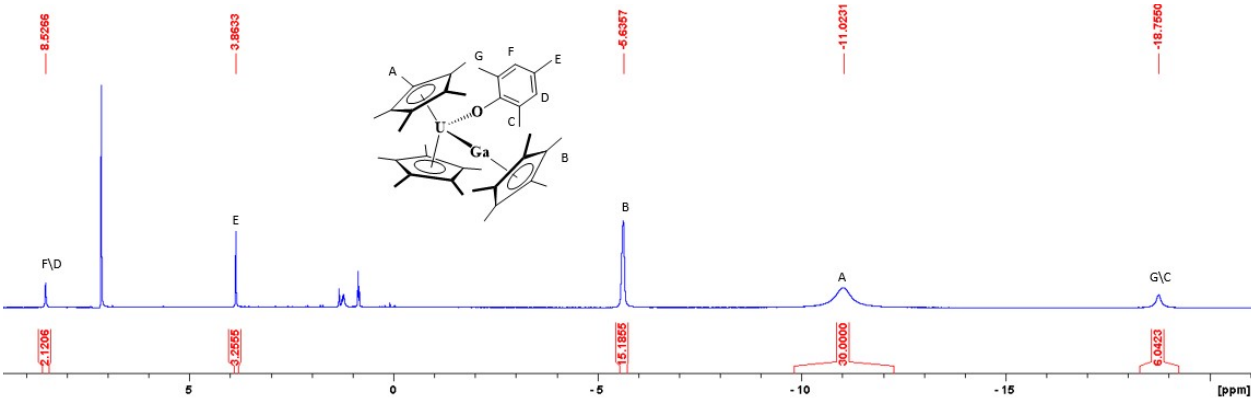

Figure S4: NMR spectrum of $[(C_5Me_5)_2(MesO)UGa(C_5Me_5)]$ in benzene-d_6.

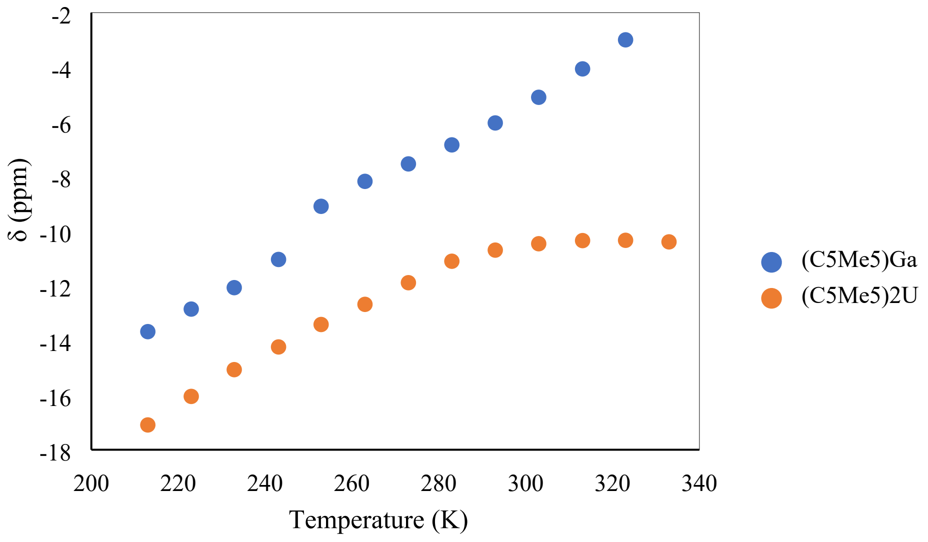

Figure S5: Plot of the ^{1}H NMR chemical shift (ppm) versus temperature (K) of $[(C_5Me_5)_2(MesO)UGa(C_5Me_5)]$, **3**, with $[(C_5Me_5)Ga]$ fragment in blue and $[(C_5Me_5)_2U]$ fragment in orange.

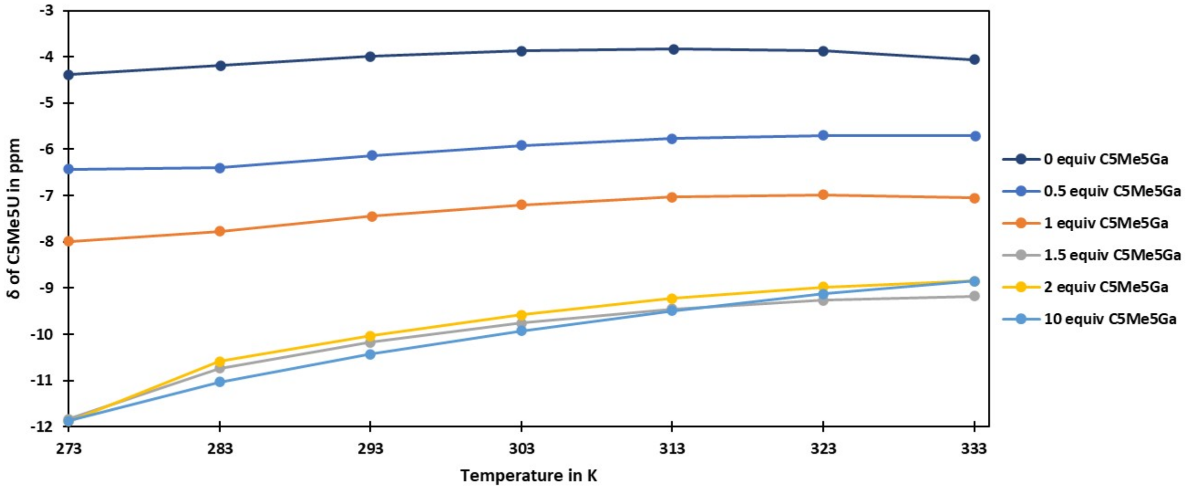

Figure S6: Plot of ^{1}H NMR chemical shift (ppm) versus temperature (K) for the $(C_5Me_5)_2U$ protons in the $[(C_5Me_5)_2(MesO)UGa(C_5Me_5)]$, **3**, system with n equivalents of $[(C_5Me_5)Ga]$, in d_8-toluene.

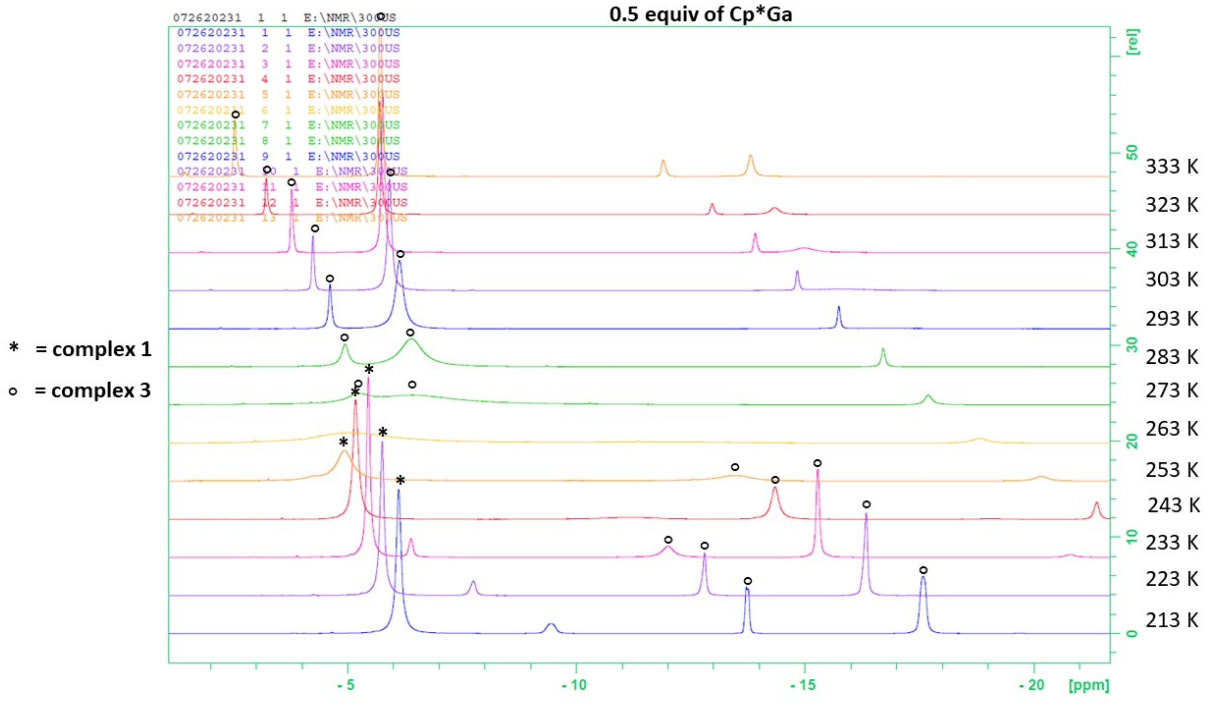

Figure S7. Variable temperature ^{1}H NMR spectrum of reaction of **1** with $[(C_5Me_5)Ga]$.

Synthesis of [(C_5Me_5)$_2$(MesO)U{μ-H_3Al(C_5Me_5)}], 4, method 1: To a pentane solution (10 mL) of [(C_5Me_5)$_2$(MesO)U(H)] (0.0391 g, 0.0607 mmol), crystalline [(C_5Me_5)AlH_2]$_3$ was added (0.0100 g, 0.0203 mmol). Over the course of a few minutes a red powder precipitates from the solution, which was cooled to -45 °C, to further precipitate [(C_5Me_5)$_2$(MesO)U{μ-H_3Al(C_5Me_5)}] (16.3 mg, 0.0202 mmol, 33%). However, the NMR spectrum of the mother liquor showed complete conversion to [(C_5Me_5)$_2$(MesO)U{μ-H_3Al(C_5Me_5)}]. ^{1}H NMR (C_6D_6, 298 K, ppm): δ -17.65 (s, 3H, o-Ar-C**H_3**), -11.32 (s, 3H, o-Ar-C**H_3**), -4.12 (s, 15H, Al(C_5(C**H_3**)$_5$), 1.68 (s, 3H, p-Ar-C**H_3**), 4.21 (s, 1H, m-Ar-**H**), 6.11 (s, 1H, m-Ar-**H**), 7.37 (s, 30H, U(C_5(C**H_3**)$_5$) hydride peaks were not located. IR (KBr, cm^{-1}): 2907 (s), 2856 (m), 1618 (bw), 1473 (m), 1385 (vs), 1229 (s), 1160 (m), 1048 (bs), 820 (s), 519 (w), 485 (w). Anal. Calcd. theory (found) for $C_{39}H_{59}$UOAl, C 57.91 (57.86%), H 7.35 (7.64%).

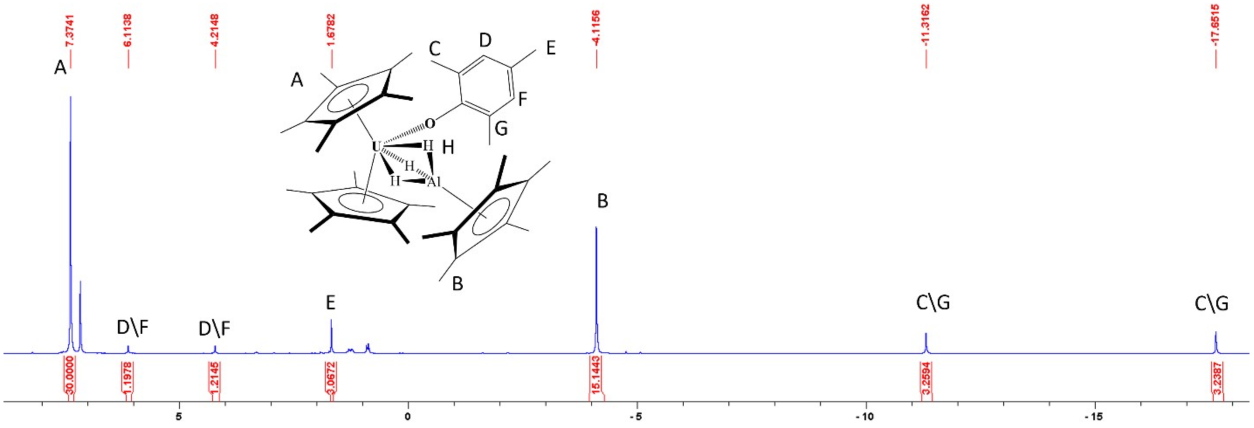

Figure S8: ^{1}H NMR spectrum of [(C_5Me_5)$_2$(MesO)U{μ-H_3Al(C_5Me_5)}] in benzene-d_6.

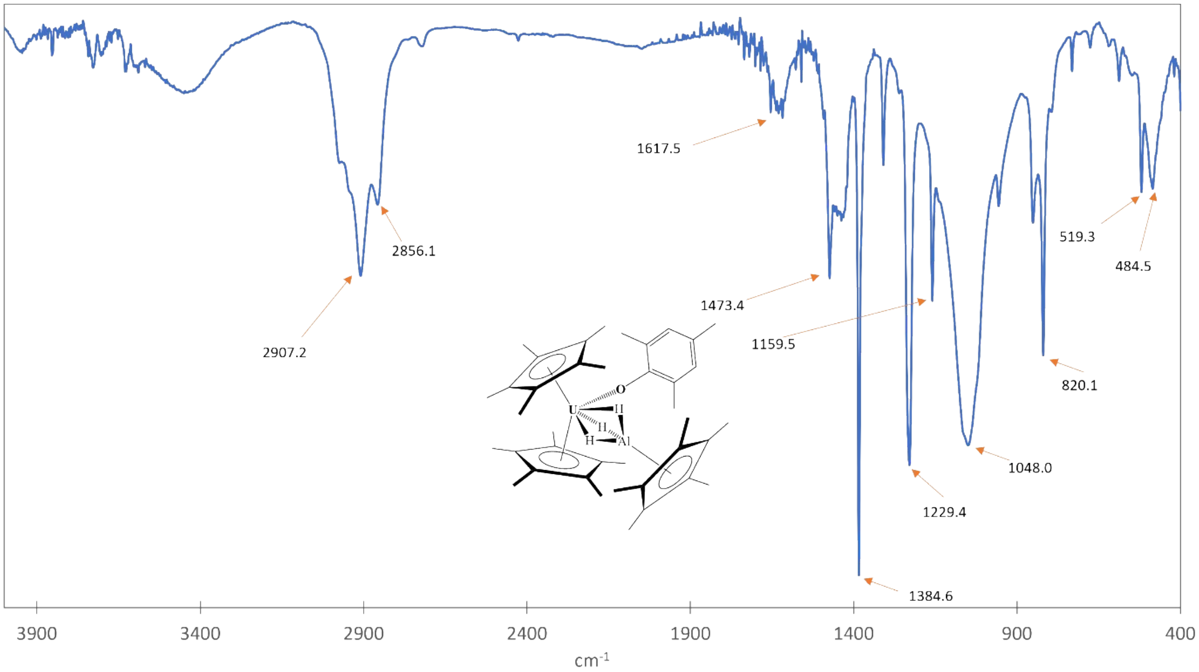

Figure S9: FTIR vibrational spectrum of [$(C_5Me_5)_2$(MesO)U{μ-H_3Al(C_5Me_5)}].

[$(C_5Me_5)_2$(MesO)U{*μ*-H_3Al(C_5Me_5)}], method 2: [$(C_5Me_5)_2$(MesO)UAl(C_5Me_5)] was dissolved in a valved NMR tube in C_6D_6 making a green solution. To this solution hydrogen was added (760 torr). The solution was heated to 50 °C for five hours then left at room temperature for 36 hours, yielding a red solution. Examination of the NMR spectrum showed a solution which had completely converted to, by integration, [$(C_5Me_5)_2$(MesO)U{*μ*-H_3Al(C_5Me_5)}] (63%), [$(C_5Me_5)_2$(MesO)U(H)] (37%), and [(C_5Me_5)AlH_2]$_3$ which had partially precipitated.

[$(C_5Me_5)_2$(MesO)U{*μ*-H_3Al(C_5Me_5)}], method 3: [$(C_5Me_5)_2$(MesO)U(THF)] (0.1198 g, 0.1676 mmol) was dissolved in toluene (8 mL). To the dark green solution [(C_5Me_5)AlH_2]$_3$ (0.0412 g, 0.0836 mmol) was added. After stirring for an hour room temperature, the solution turned black. The only isolable product, by crystallization from pentane at -16 °C, of the reaction was [$(C_5Me_5)_2$(MesO)U{*μ*-H_3Al(C_5Me_5)}] (0.0058 mg, 4%). In the NMR of the crude product [(C_5Me_5)Al]$_4$ is observed along with another likely uranium containing product.

Synthesis of [$(C_5Me_5)_2$(MesO)U(H)], 5, method 1: [$(C_5Me_5)_2$(MesO)U(THF)], **1**, (0.3880 g, 0.5426 mmol) was dissolved in 15 mL of toluene, in a 100 mL bomb flask. To this solution dihydrogen was added (760 torr). The solution was heated to 65 °C and stirred overnight, yielding a dark red solution of [$(C_5Me_5)_2$(MesO)U(H)], **5**, and [$(C_5Me_5)_2$(MesO)U(O^nBu)], the next morning. Volatiles were removed under reduced pressure, and the dark orange oil was redissolved a minimal amount of hexamethyldisiloxane (~4 mL). This solution was cooled to -45 °C, yielding [$(C_5Me_5)_2$(MesO)U(H)] (0.0839 g, 0.1301 mmol, 24%). ^{1}H NMR (C_6D_6, 298 K, ppm): δ -30.81 (s, 3H, o-Ar-C**H$_3$**), -28.63 (s, 3H, o-Ar-C**H$_3$**), 2.86 (s, 3H, p-Ar-C**H$_3$**), 3.32 (s, 30H, U(C_5(C**H$_3$**)$_5$)$_5$), 9.50 (s, 2H, m-Ar**H**), 272.75 (U-**H**). IR (KBr, cm^{-1}): 2936 (m), 2905 (s), 2856 (m), 1472 (s), 1384 (s), 1309 (m), 1233 (vs), 1157 (s), 1082 (m), 835 (s), 729 (w), 531 (m). Anal. Calcd. theory (found) for $C_{29}H_{42}$UO, C 54.03 (54.16%), H 6.57 (6.20%).

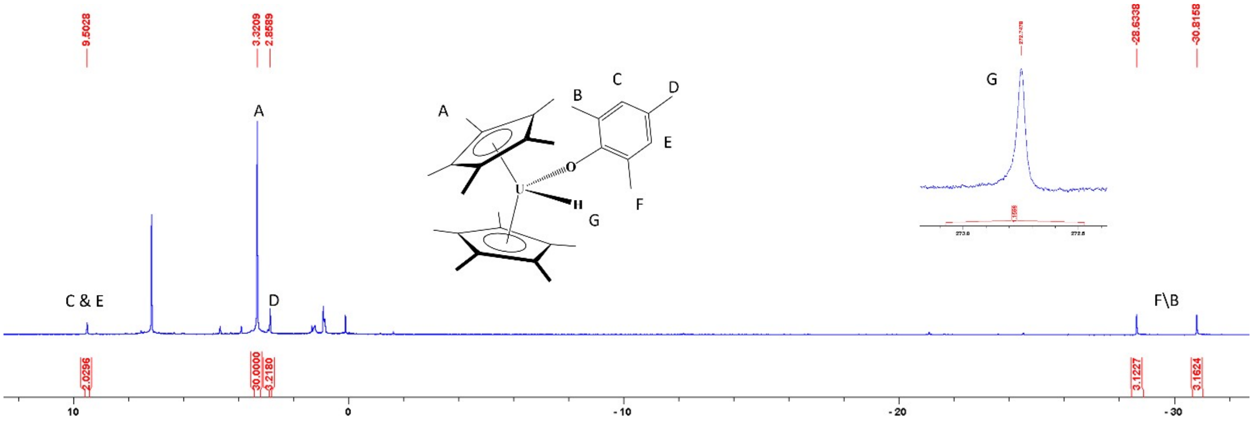

Figure S10: ^{1}H NMR spectra of $[(C_5Me_5)_2(MesO)U(H)]$ in benzene-d_6.

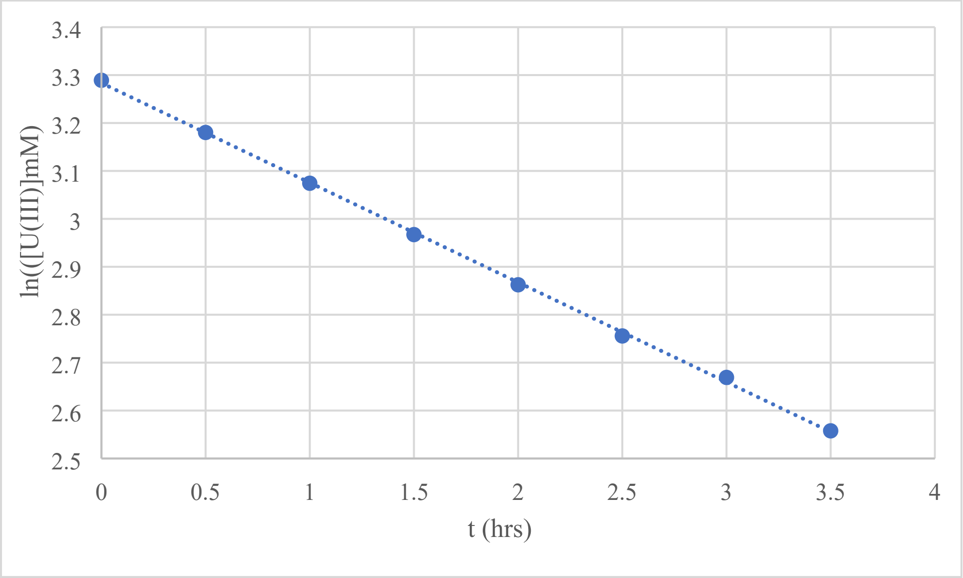

Figure S11: First order kinetics graph for the reaction of $[(C_5Me_5)_2(MesO)U(THF)]$ with H_2.

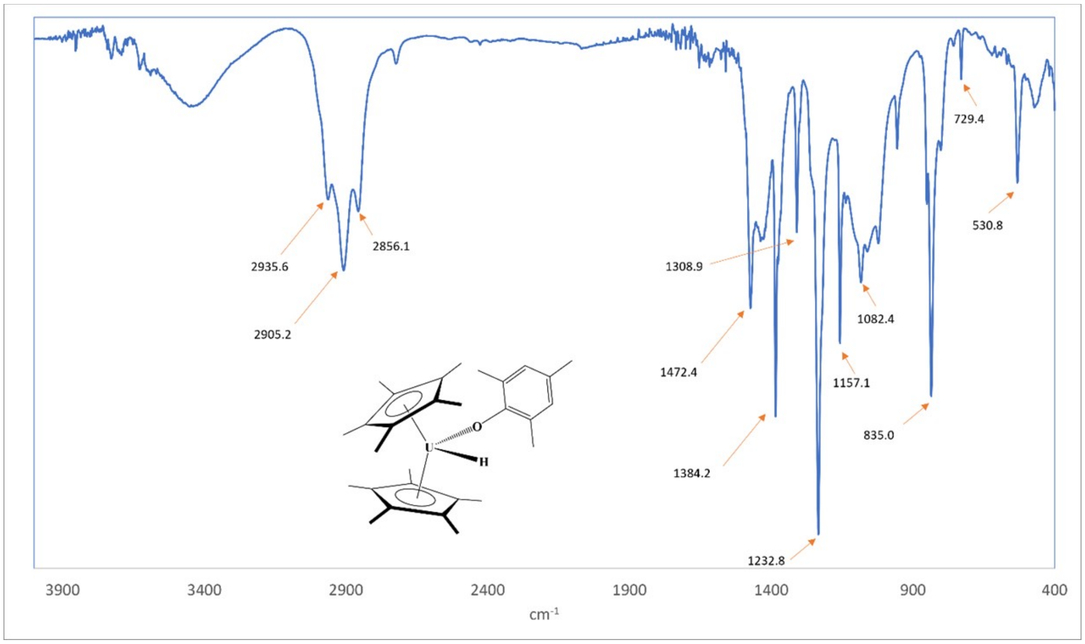

Figure S12: FTIR vibrational spectrum of [$(C_5Me_5)_2$(MesO)U(H)].

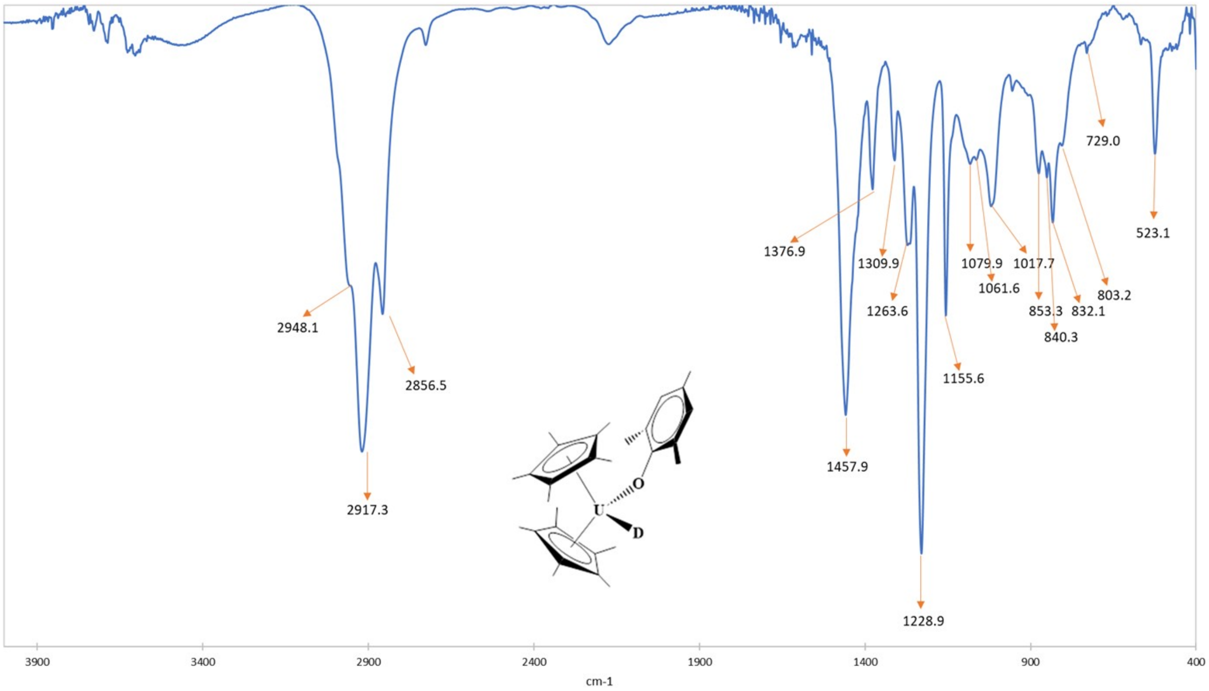

Figure S13: FTIR vibrational spectrum of [$(C_5Me_5)_2$(MesO)U(D)].

[$(C_5Me_5)_2$(MesO)U(H)], method 2: To a valved NMR tube [$(C_5Me_5)_2$(MesO)UGa(C_5Me_5)] dissolved in C_6D_6 and hydrogen were added (1 atm). The solution was heated to 65 °C for 36 hr, which converted to, by integration [$(C_5Me_5)_2$(MesO)U(H)] (13%), C_5Me_5H was also observed. A grey precipitate, presumably gallium metal, was observed in the bottom of the NMR tube.

Independent synthesis of [(C_5Me_5)$_2$(MesO)U(O-^{n}Bu)], 6: [(C_5Me_5)$_2$(MesO)U(I)] (0.0671g, 0.0871 mmol) was dissolved in diethyl ether (6 mL). To this solution potassium *n*-butoxide was added (0.0117 g, 0.1043 mmol). The solution quickly turned from red to cloudy yellow, and the reaction was allowed to stir for two hours, after which volatiles were removed under reduced pressure. The powder was extracted with hexamethyldisiloxane (3 mL). Yellow crystals were grown by slow evaporation of the HMDSO solution (0.0301 g, 0.0420 mmol, 48%). ^{1}H NMR (C_6D_6, 298 K, ppm): δ -21.08 (s, 3H, o-Ar-C**H$_3$**), -1.62 (s, 3H, o-Ar-C**H$_3$**), 0.19 (s, 1H, m-Ar-**H**), 0.91 (s, 30H, U(C_5(C**H**$_3$)$_5$)), 2.94 (s, 3H, p-Ar-C**H**$_3$), 3.30 (t, 3H, CH_2-CH_2-CH_2-C**H$_3$**), 4.30 (s, 1H, m-Ar-**H**). 6.01 (q, 2H, CH_2-CH_2-C**H$_2$**-CH_3), 9.16 (s, 2H, CH_2-C**H$_2$**-CH_2-CH_3), 44.00 (s, 2H, CH_2-CH_2-CH_2-C**H$_3$**). IR (KBr, cm^{-1}): 2957 (m), 2925 (sb), 2858 (m), 1471 (m), 1436 (wb), 1385 (vs), 1309 (w), 1240 (s), 1129 (w), 1082 (vs), 831 (m), 522 (w). Anal. Calcd. theory (found) for $C_{33}H_{50}UO_2$, C 55.30 (55.66%), H 7.03 (7.36%).

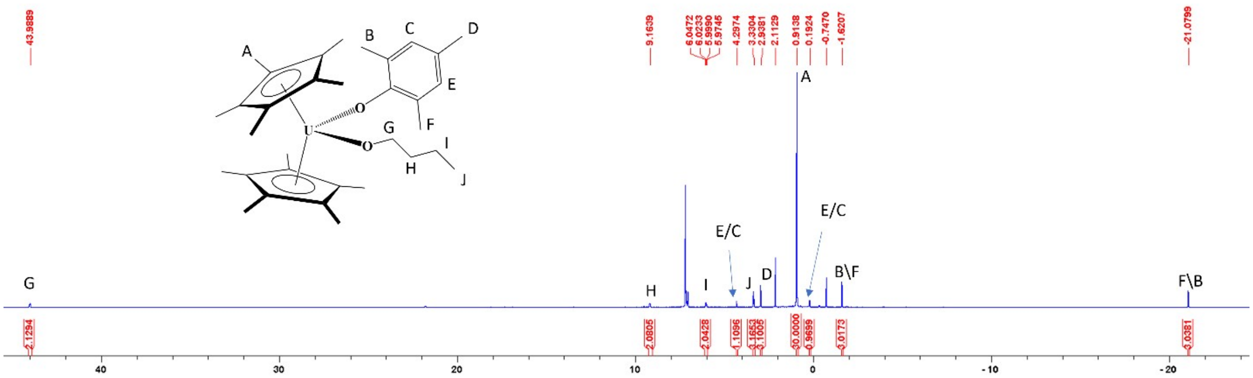

Figure S14: ^{1}H NMR spectrum of [(C_5Me_5)$_2$(MesO)U(O-^{n}Bu)], **6**.

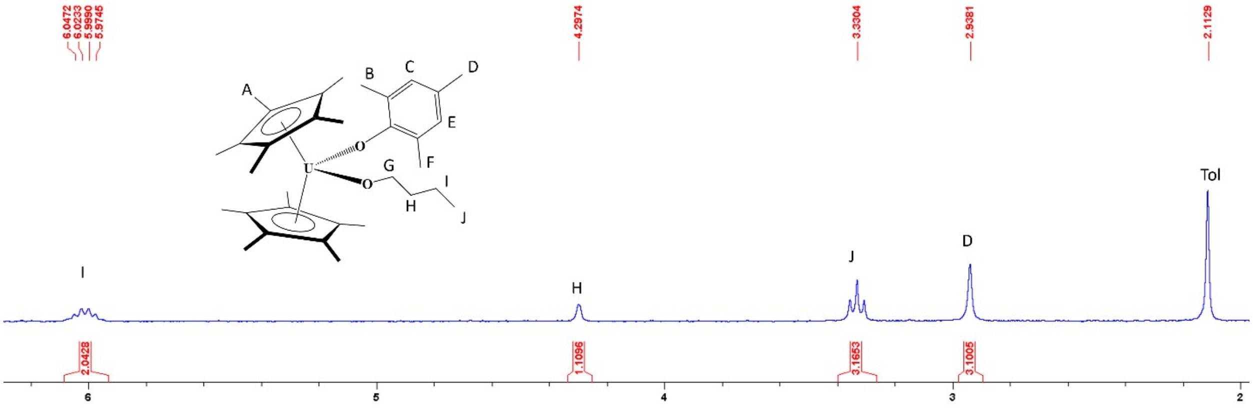

Figure S15: Zoomed in ^{1}H NMR spectrum of [(C_5Me_5)$_2$(MesO)U(O-^{n}Bu)].

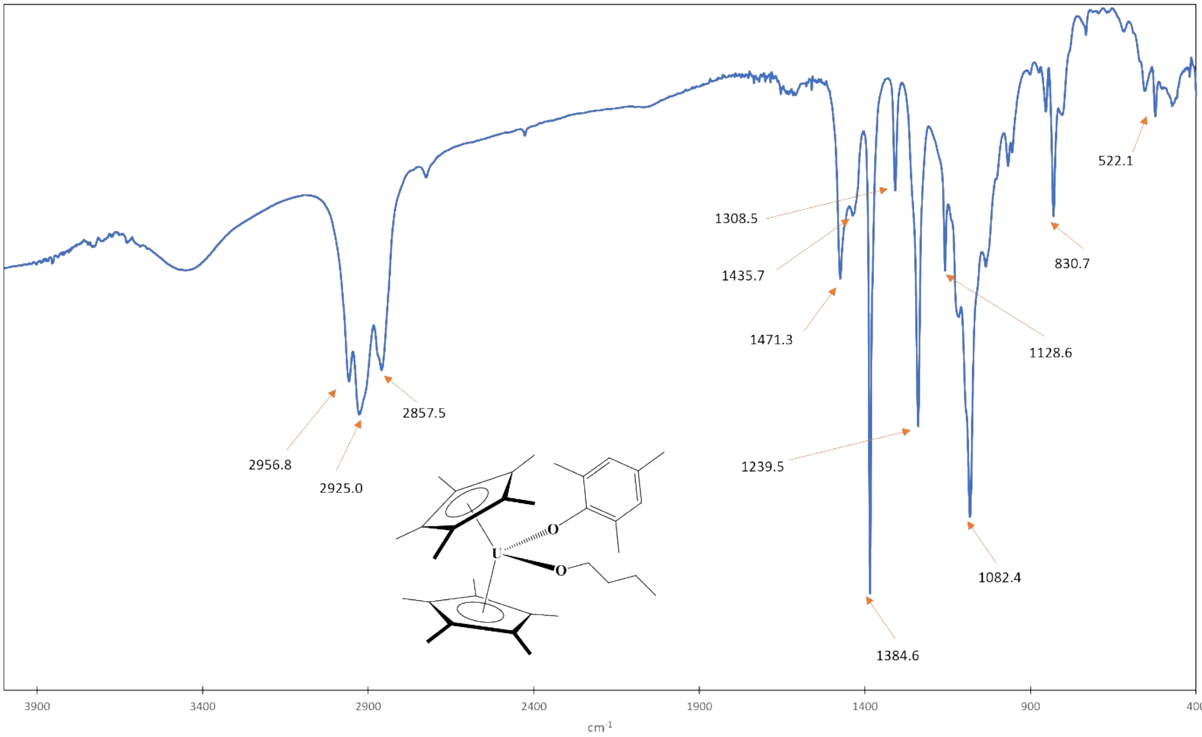

Figure S16: FTIR vibrational spectrum of [(C_5Me_5)$_2$(MesO)U(O-^{n}Bu)].

Synthesis of [(C_5Me_5)$_2$(MesO)U(I)], 7: [(C_5Me_5)$_2$(MesO)U(THF)] (0.1121 g, 0.1568 mmol) was dissolved in pentane (15 mL) and cooled to -45 °C, when iodine (0.0199 g, 0.0784 mmol) dissolved in pentane (5 mL) was added. The solution immediately turned red, volatiles were removed under reduced pressure, and the solution was recrystallized from diethyl ether, cleanly yielding [(C_5Me_5)$_2$(MesO)U(I)] (0.0582 g, 0.0755 mmol, 48%). ^{1}H NMR (C_6D_6, 298 K, ppm): δ -24.56 (s, 3H, o-Ar-C**H$_3$**), -12.16 (s, 3H, o-Ar-C**H$_3$**), 6.34 (s, 3H, p-Ar-C**H**$_3$),8.09 (s, 1H, m-Ar-**H**), 9.48 (s, 30H, U(C_5(C**H**$_3$)$_5$) 11.16 (s, 1H, m-Ar-**H**). IR (KBr, cm^{-1}): 2969 (m), 2904 (s), 2854 (s), 1474 (s), 1432 (mb), 1385 (s), 1310 (w), 1222 (vs), 1157 (s), 1098 (mb), 1020 (m), 840 (m), 727 (w), 534 (m). Anal. Calcd. theory (found) for $C_{29}H_{41}UOI$, C 45.20 (45.45%), H 5.36 (5.28%).

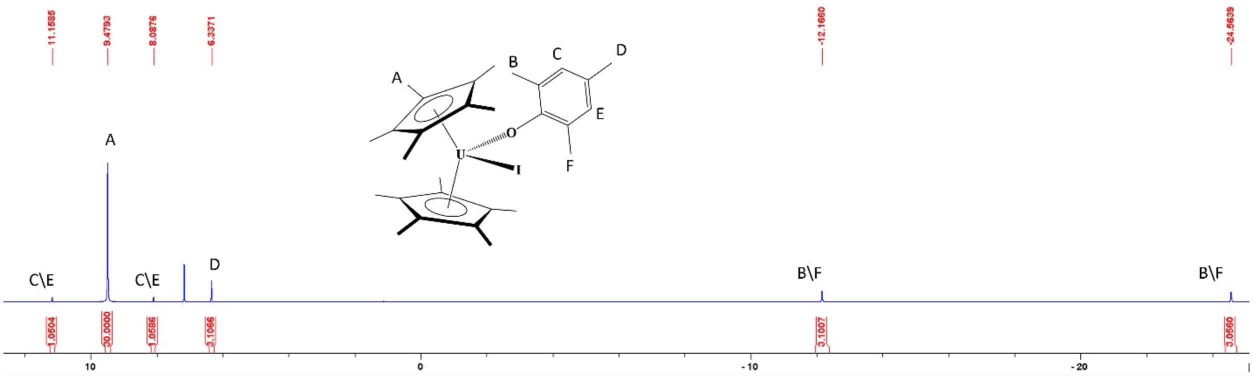

Figure S17: ^{1}H NMR spectrum of [(C_5Me_5)$_2$(MesO)U(I)] in benzene-d_6.

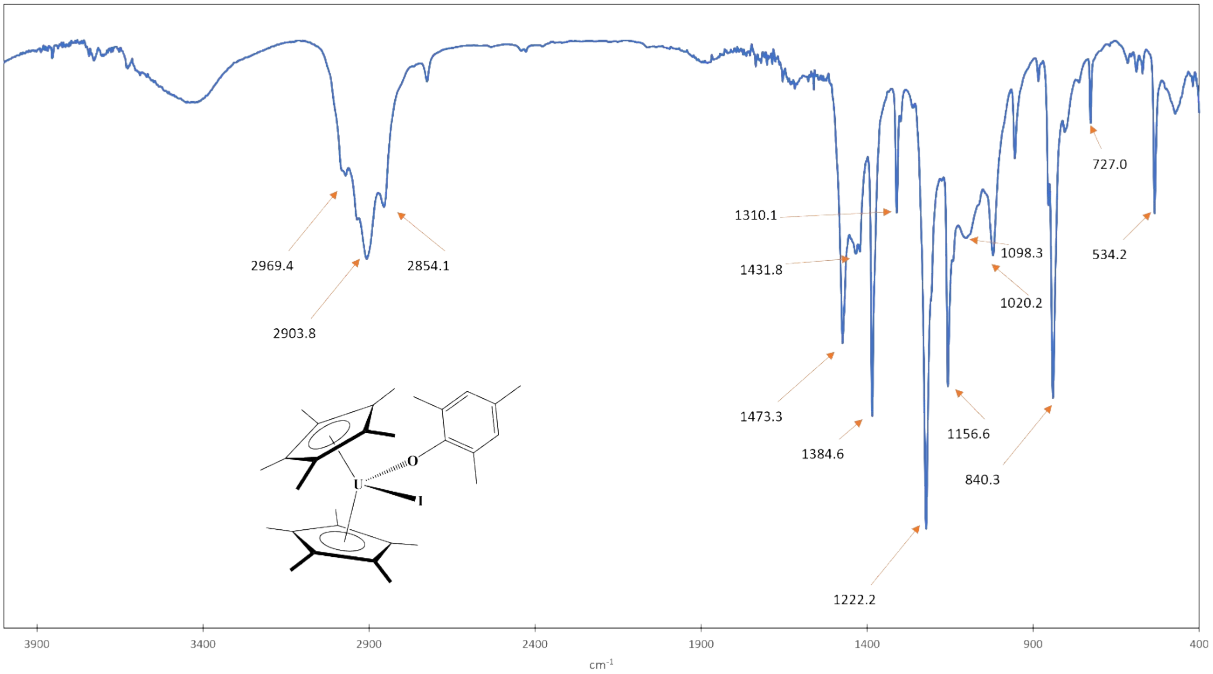

Figure S18: FTIR vibrational spectrum of [(C_5Me_5)$_2$(MesO)U(I)].

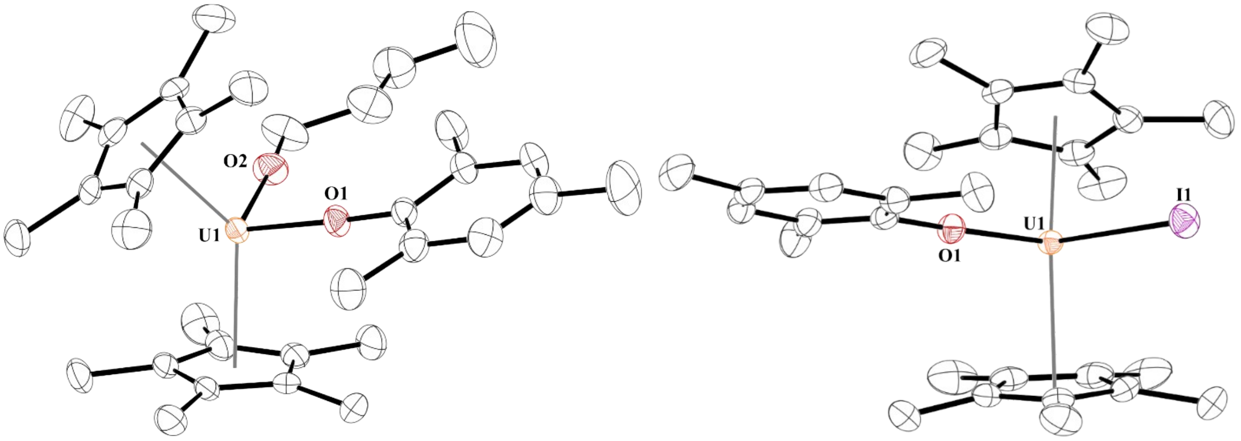

Figure S19: Thermal ellipsoid of **6** (left) and **7** (right) shown at the 50% probability level. The hydrogen atoms have been omitted for clarity.

Synthesis of $(C_5Me_5)_2(MesO)U(CH_2CH_3)$, 8. To a stirring -45°C solution of $[(C_5Me_5)_2(MesO)U(THF)]$, 114 mg (0.160 mmol), in pentane (20 mL), diethylzinc 10 mg, 0.081 mmol) was added. As the solution warmed to room temperature the solution turned red. After an hour the vial is plated with zinc metal. The solution was filtered and volatiles were removed under reduced pressure yielding 101 mg (0.151 mmol, 94%). ^{1}H NMR (C_6D_6, 298 K): -182.76 (s, 2H, C**H**$_2$CH$_3$), -15.53 (s, 3H, o-Ar- C**H**$_3$), -9.81 (s, 3H, CH$_2$C**H**$_3$), -6.86 (s, 3H, o-Ar-C**H**$_3$), 4.31 (s, 30H, C_5(C**H**$_3$)$_5$), 5.44 (s, 3H, p-Ar-C**H**$_3$), 9.26 (s, 1H, m-Ar-**H**), 11.40 (s, 1H, m-Ar-**H**). IR (KBr, cm^{-1}): 2963 (m), 2905 (s), 2852 (m), 1474 (m), 1430 (m), 1384 (m), 1309 (m), 1262 (s), 1232 (s), 1093 (sb), 1021 (s), 832 (s), 802 (s), 527 (m), 463 (m). Anal. Calcd. theory (found) for $UOC_{31}H_{46}$, C 53.35 (55.06%), H 6.89 (6.95%).

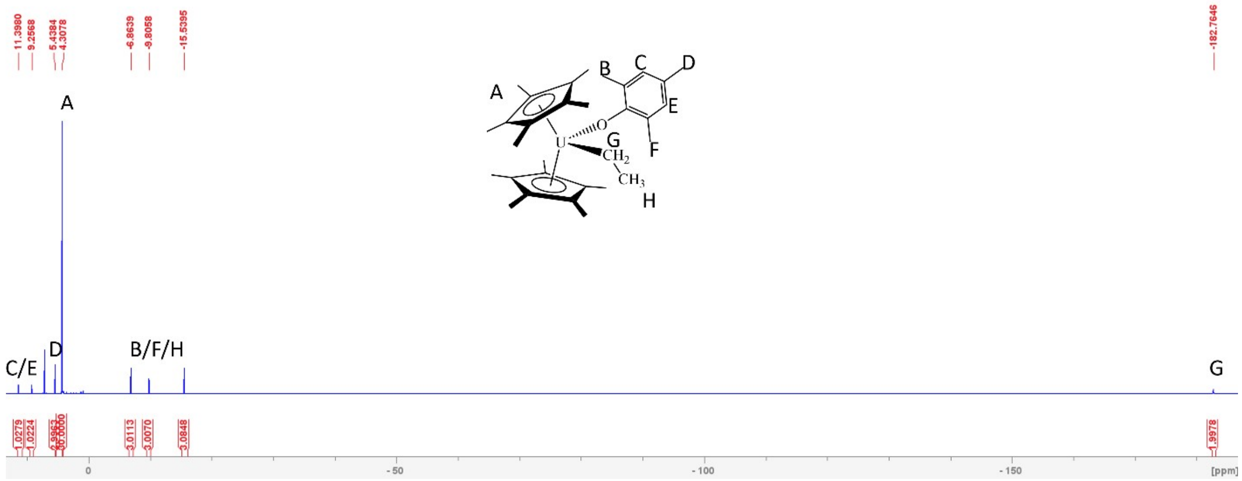

Figure S20: ^{1}H NMR spectrum of $[(C_5Me_5)_2(MesO)U(CH_2CH_3)]$.

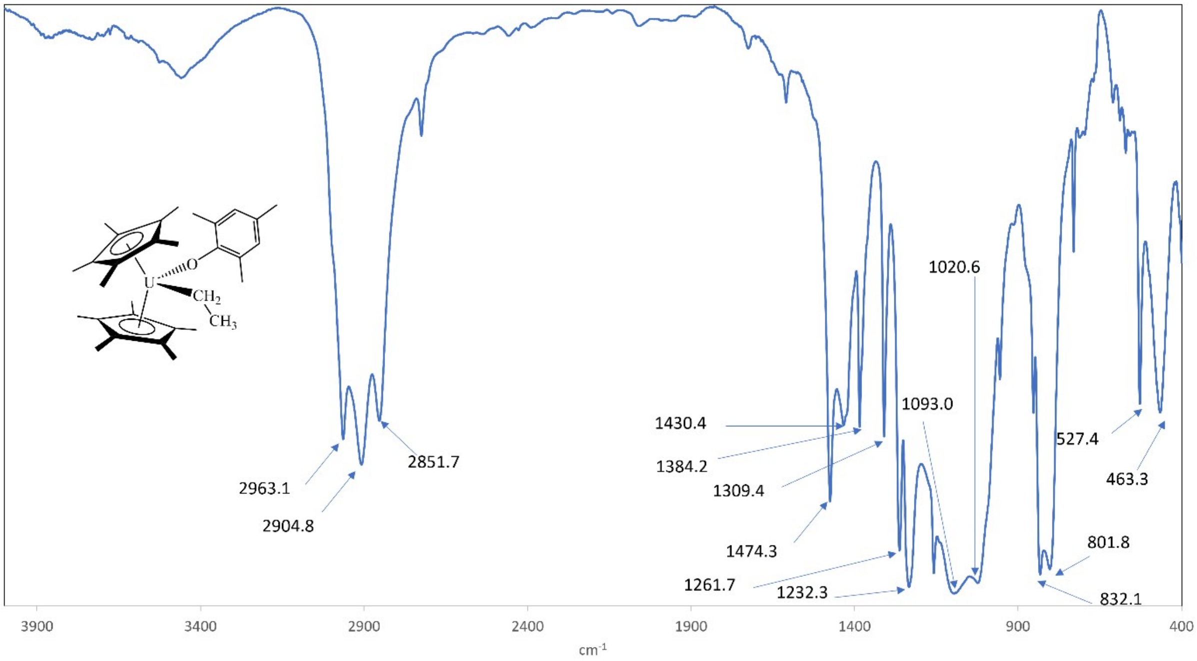

Figure S21: FTIR vibrational spectrum of [$(C_5Me_5)_2$(MesO)U(CH_2CH_3)].

Synthesis of [$(C_5Me_5)_2$(MesO)U(H)] from **[$(C_5Me_5)_2$(MesO)U(CH_2CH_3)] and H_2:** To a J. Young tube charged with [$(C_5Me_5)_2$(MesO)U(Et)] and deuterated benzene, the atmosphere was replaced with hydrogen (760 torr). After sitting at room temperature for 12 hrs, the sample has quantitatively converted to [$(C_5Me_5)_2$(MesO)U(H)].

Synthesis of [$(C_5Me_5)_2$(MesO)U(CH_3)], 9. [$(C_5Me_5)_2$U(I)(CH_3)] (150.0 mg, 0.23 mmol) was dissolved in 5 mL of toluene and combined with [KOMes] (40.0 mg, 0.23 mmol) in 5 mL of toluene. The combined toluene solutions were stirred for 16 hours. The resulting orange solution was filtered through Celite and volatiles were removed, under reduced pressure, yielding 126.0 mg [$(C_5Me_5)_2$U(OMes)(CH_3)] (0.19 mmol, 83%). X-ray quality crystal can be grown from pentane and toluene mixture stored at -14 °C. ^{1}H NMR (C_6D_6, 300K): -183.1 (s, 3H, U-C**H**$_3$), -18.60 (s, 3H, o-Ar-C**H**$_3$), -13.30 (s, 3H, o-Ar-C**H**$_3$), 4.41 (bs, 30H, U(C_5(C**H**$_3$)$_5$), 4.88 (s, 3H, p-Ar-C**H**$_3$), 8.06 (s, 1H, m-Ar-**H**). 9.78 (s, 1H, m-Ar-**H**). IR (KBr, cm^{-1}): 2968 (s), 2911 (s), 2859 (s), 1474 (s), 1437 (m), 1378 (m), 1310 (m), 1235 (vs), 1158 (s), 1106 (m), 1021 (m), 956 (w), 853 (m), 837 (s), 802 (w), 730 (w), 531 (m). Anal. Calcd. theory (found) for $C_{30}H_{44}UO$, C 54.70 (54.36%), H 6.73 (6.34%).

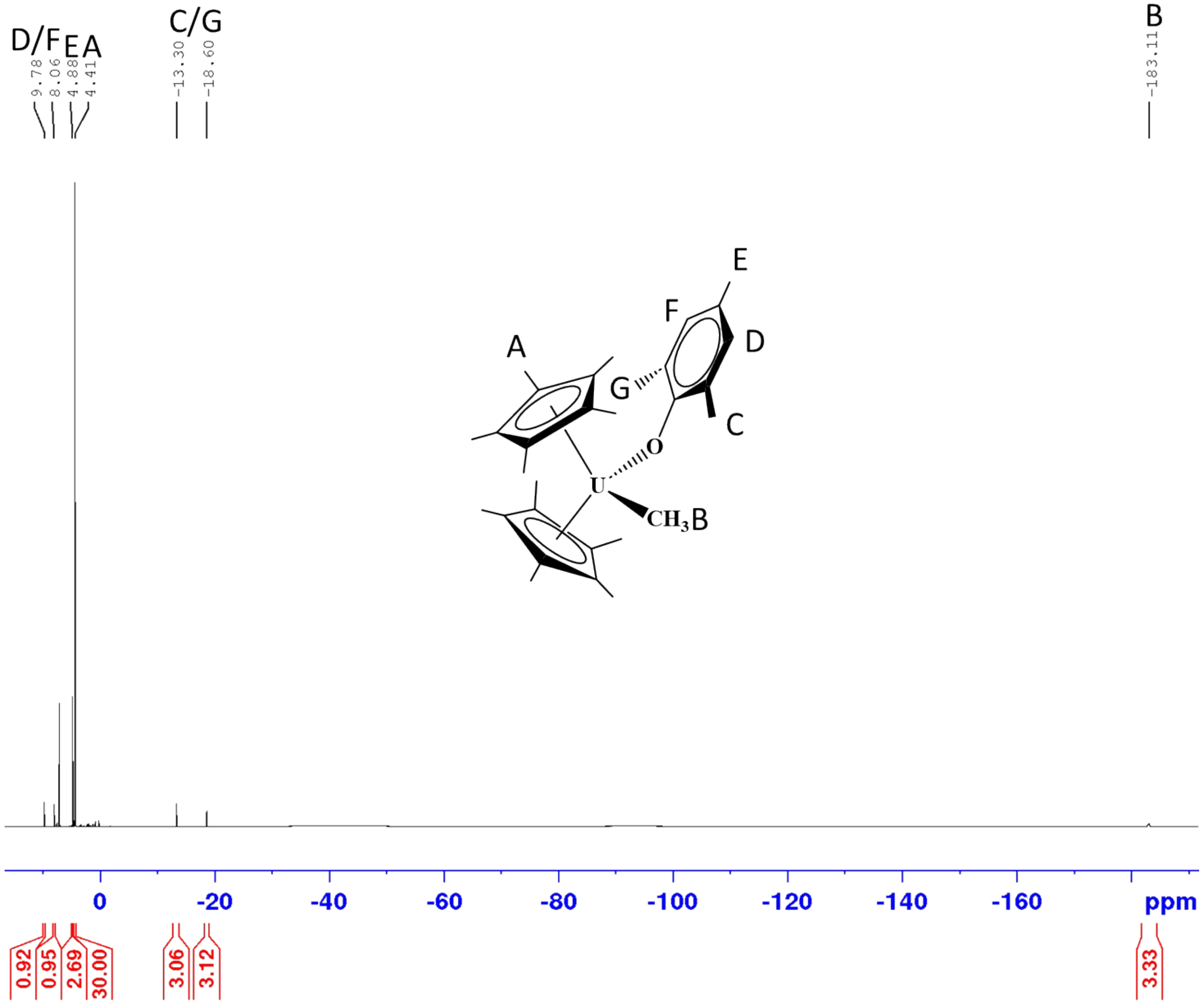

Figure S22: ^{1}H NMR spectrum of $[(C_5Me_5)_2(MesO)U(CH_3)]$ in benzene-d_6.

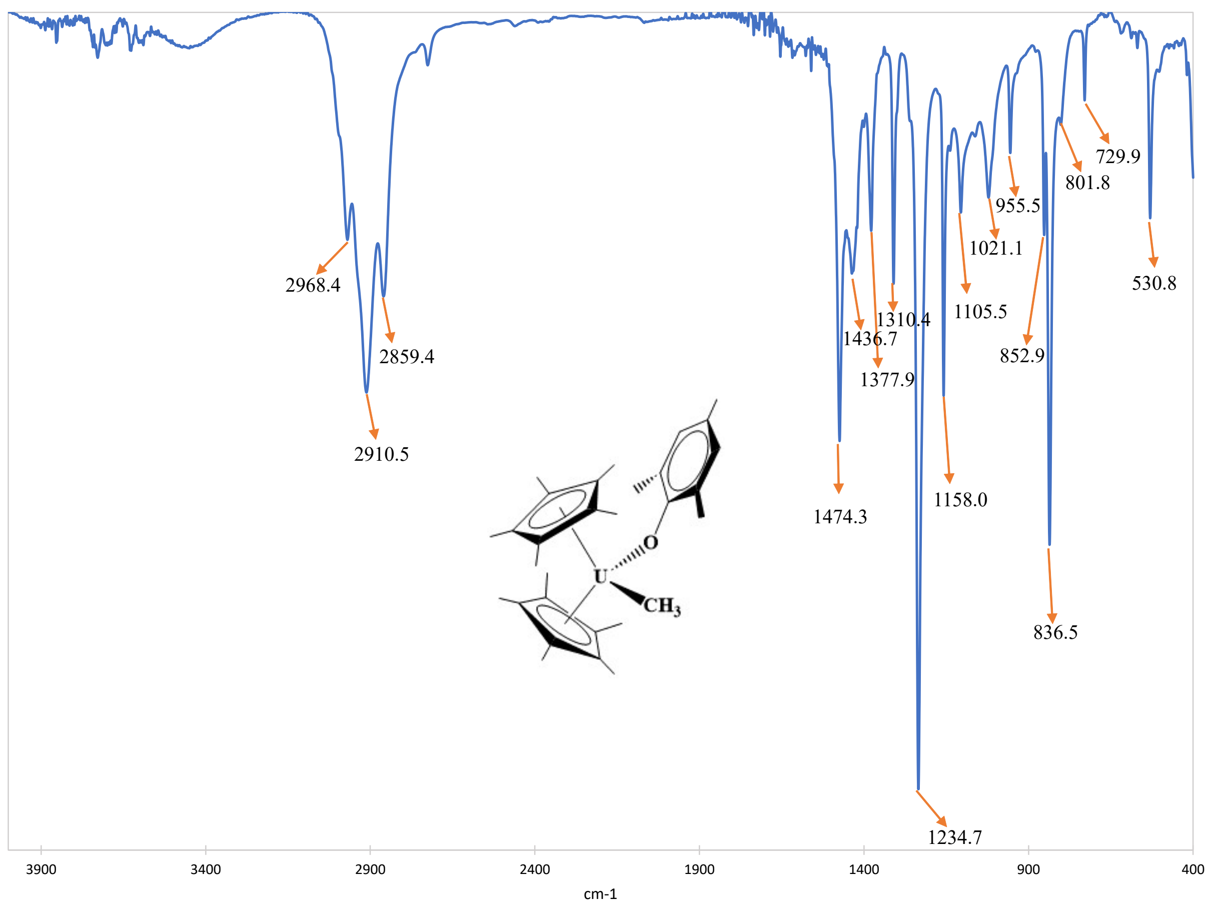

Figure S23: FTIR vibrational spectrum of [$(C_5Me_5)_2$(MesO)U(CH_3)].

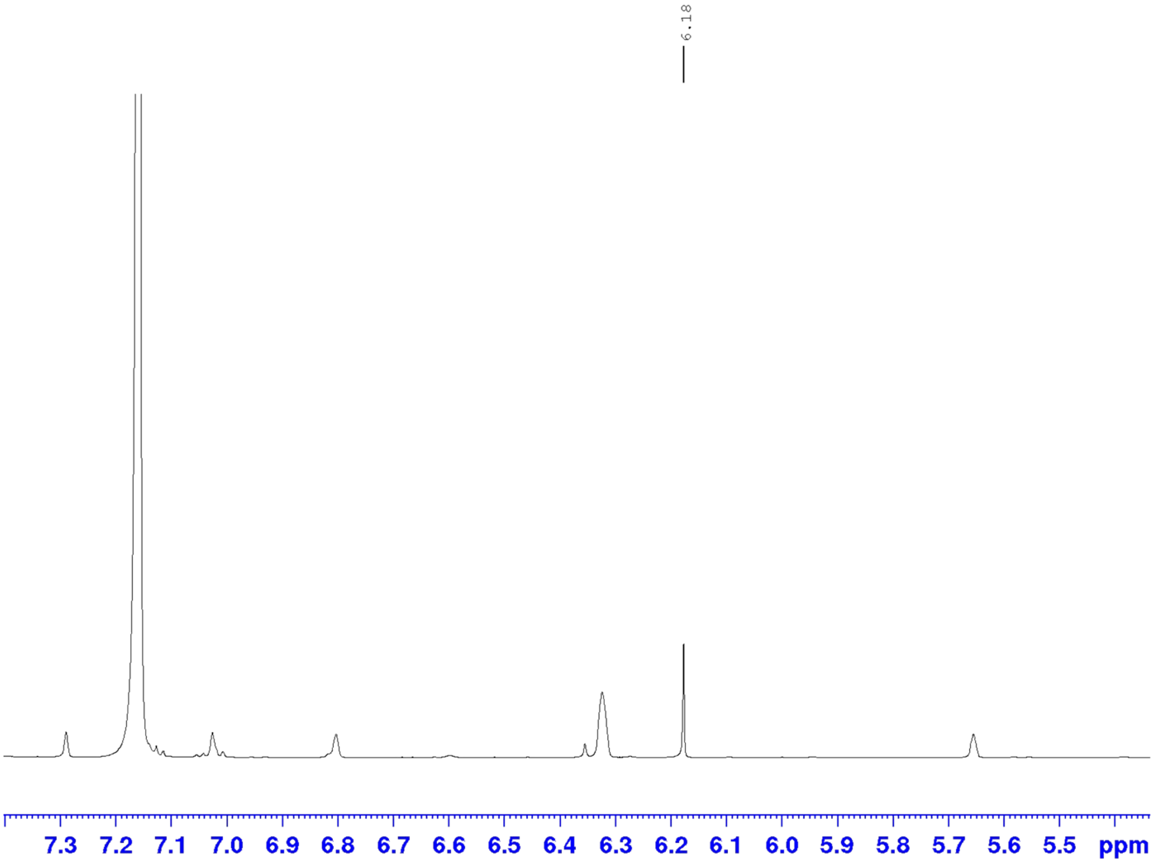

Figure S24: Detection of $CHCl_3$ (6.18 ppm) from [$(C_5Me_5)_2$(MesO)U(H)] and CCl_4 in C_6D_6.

Crystal Structure Refinement Details: Single crystal X-ray diffraction (SCXRD) data for **2** was measured on a Bruker X8 Prospector diffractometer equipped with an Apex II CCD area detector using Cu-Kα radiation from a microfocus source (Bruker AXS, Madison, WI, USA). SCXRD data for **3**, **4**, **5**, **6**, **8**, and **9** were collected on a Bruker D8 Venture diffractometer equipped with a Photon II CMOS area detector using Mo-Kα radiation from a microfocus source (Bruker AXS). SCXRD data for **7** was collected on a Bruker SMART diffractometer equipped with an Apex II detector using Mo-Kα radiation from a sealed source with focusing optics. All crystals were cooled to their collection temperatures under streams of cold N_2 gas using Cryostream 700 and 800 cryostats (Oxford Cryosystems, Oxford, UK). A unit cell for compound **5** was found by a least-squares search against 376 reflections with $I/\sigma > 20$; of these 101 could be indexed to integer Miller indices against which the cell parameters were refined to the following values:

Triclinic P, a = 8.49(2) Å, b = 9.83(2) Å, c = 17.46(3) Å; α = 81.36(8)°, β = 89.34(7)°, γ = 70.09(7)°

For all other crystals, hemispheres of unique data were collected using strategies of scans about the omega and phi axes with 0.5° frame widths. Unit cell determination, data collection, data reduction, absorption correction and scaling, and space group determination were done using the Bruker Apex4 software suite.[7]

The structures of **2**, **3**, **4**, **6**, **7**, and **8** were solved by an iterative dual-space method as implemented in SHELXT.[8] **9** solved by direct methods as implemented in SHELXS.[9] All structures were refined by full-matrix least squares refinement against F^2 using SHELXL,[10] using Olex2 as an interface for model building and visualization.[11] Full occupancy non-hydrogen atoms were refined anisotropically without restraints. For structure **6** the thermal parameters for the minor conformer of the disordered butanoate ligand were restrained to show rigid bond behavior.[12] For structure **9** the minor conformer of the disordered Cp* ligand was refined isotropically. Hydrogen atoms bonded to carbon were placed in calculated positions with their thermal parameters and coordinates constrained to ride on the carrier atoms; methyl group hydrogen atoms were allowed to rotate about the C-C bond axis with fixed H-C-H angles. Hydride ions in **4** were located from the difference map, and their coordinates were freely refined while their thermal parameters were constrained to ride on the Al atom.

The structure of **2** contains difference map artifacts which are caused by inadequate correction for absorption. The estimated minimum beam transmission is 0.8%; at these near-zero values small imprecisions in the estimated value have larger effects on the model. The structure of **8** refined to have difference map artifacts including both negative peaks near the U atoms and large positive peaks in chemically unrealistic positions. The peaks correspond to the projection of the U atoms of the symmetry-inequivalent molecules by a half translation along b, indicating they are caused by crystallographic defects where the molecules substitute each other. The minor peaks were refined as U atoms at 3% occupancy, the major U atoms were fixed at 97% occupancy. Only the U atom can be located and refined at this low occupancy, so all other atoms were refined at 100% occupancy using the PART instruction to remove superfluous bonds to the minor U atom.

Table S1. X-ray crystallographic details for complexes **2-4** and **6**.

	**2**	**3**	**4**	**6**
**CCDC deposit number**	2264161	2264162	2264163	2264164
**Chemical formula**	$3(C_{39}H_{56}AlOU)\bullet C_7H_8$	$C_{39}H_{56}GaOU$	$C_{39}H_{59}AlOU$	$C_{33}H_{50}O_2U$
**Formula weight (g/mol)**	2509.67	848.58	808.87	716.76
**Crystal habit/color**	Block/Brown	Block/Black	Plate/Brown	Fragment/Orange
**Temperature (K)**	170(2)	100(2)	173(2)	173(2)
**Space group**	*P*121/m1	*P*-1	*P*-1	*P*-1
**Crystal system**	Monoclinic	Triclinic	Triclinic	Triclinic
**Volume (Å)**	5802.3(2)	1814.2(6)	1785.19(13)	1567.1(2)
**a (Å)**	11.7527(3)	9.6007(18)	9.6070(4)	10.0523(8)
**b (Å)**	28.5642(6)	12.037(2)	11.8205(5)	11.2703(9)
**c (Å)**	17.9883(4)	16.215(3)	16.2324(6)	14.4867(11)
**α (deg)**	90	97.253(6)	96.772(2)	91.227(3)
**β (deg)**	106.0880(10)	97.094(6)	97.569(2)	106.016(3)
**γ (deg)**	90	99.358(7)	99.253(2)	95.893(3)
**Z**	2	2	2	2
**Calculated density (g/cm^3)**	1.436	1.553	1.505	1.519
**Absorption coefficient (mm^{-1})**	12.231	5.227	4.599	5.204
**Final *R* indices [$I > 2\sigma(I)$]**	$R = 0.0609$	$R = 0.0459$	$R = 0.0364$	$R = 0.0336$
	$R_w = 0.1611$	$R_w = 0.0999$	$R_w = 0.0529$	$R_w = 0.0754$

Table S2. X-ray crystallographic details for complexes **7-9**.

	**7**	**8**	**9**
**CCDC deposit number**	2264165	2264166	2264167
**Chemical formula**	$C_{29}H_{41}IOU$	$C_{31}H_{46}OU$	$C_{30}H_{44}OU$
**Formula weight (g/mol)**	770.55	672.71	658.68
**Crystal habit/color**	Block/Red	Needle/Orange	Tablet/Brown
**Temperature (K)**	150(2)	173(2)	173(2)
**Space group**	$P2_1/n$	P-1	$P2_1/n$
**Crystal system**	Monoclinic	Triclinic	Monoclinic
**Volume (Å)**	2782.5(7)	2818.6(7)	2769.3(4)
**a (Å)**	10.0045(14)	8.9923(12)	8.7245(7)
**b (Å)**	18.413(3)	17.002(2)	33.797(3)
**c (Å)**	15.158(2)	19.547(3)	10.1074(8)
**α (deg)**	90	108.404(4)	90
**β (deg)**	94.813(2)	95.865(4)	111.688(3)
**γ (deg)**	90	90.279(4)	90
**Z**	4	4	4
**Calculated density (g/cm^3)**	1.839	1.585	1.580
**Absorption coefficient (mm^{-1})**	6.961	5.778	5.879
**Final *R* indices [$I > 2\sigma(I)$]**	R = 0.0264	R = 0.0629	R = 0.0256
	R_w = 0.0640	R_w = 0.1354	R_w = 0.0584

Table S3. Selected bond angles (deg) in complexes **2-4**, **6-9**.

	**2, E = Al**	**3, E = Ga**	**4, E = H**	**6, E = O^{n}Bu**	**7, E = I**	**8, E = Et**	**9, E = Me**
**Cp*^{1}-U-E**	101.34(4)<br>100.96(2)	100.98(2)	108.14(1)	103.61(9)	103.386(6)	102.2(3)<br>102.4(3)	108.5(2)
**Cp*^{2}-U-E**	100.29(4)<br>100.96(2)	102.20(2)	108.77(1)	104.05(9)	103.137(7)	103.0(3)<br>102.1(3)	98.09(8)
**E-U-O**	100.4(2)<br>96.7(3)	95.9(1)	101.97(1)	102.6(1)	99.22(7)	92.2(3)<br>92.9(3)	98.5(1)
**Cp*^{1}-U-O**	107.4(2)<br>106.22(6)	107.4(1)	104.53(4)	105.26(8)	104.52(7)	106.2(2)<br>110.7(2)	106.23(6)
**Cp*^{2}-U-O**	106.0(2)<br>106.22(6)	107.1(1)	103.31(4)	103.07(8)	104.58(7)	110.7(2)<br>105.8(2)	104.86(6)
**Cp*^{1}-U-Cp*^{2}**	135.697(8)<br>137.99(1)	135.59(1)	127.134(4)	134.445(8)	136.423(6)	133.84(2)<br>134.32(2)	141.327(5)
**U distance to basal plane**	0.449(2)<br>0.419(2)	0.426(1)	0.6964(5)	0.5597(9)	0.5008(7)	0.441(2)<br>0.408(2)	0.3589(6)

Computational Details. All-electron density functional theory (DFT) calculations were carried out for the U-Al and U-Ga complexes, using the PBE approximation for exchange-correlation.[13] The calculations were performed using the ORCA quantum chemistry program package, version 5.0.2.[14] Scalar-relativistic effects were included via the zeroth order regular approximation (ZORA)[15] as implemented in ORCA. The unrestricted Kohn-Sham wavefunctions were chosen to have $M_s = 3/2$, which corresponds to high-spin U(III). For the elements H, C, O, Al, and Ga, we utilized the Ahlrichs family of polarized triple-zeta basis sets (def2-TZVP)[16] recontracted for ZORA, and Weigend's auxiliary basis sets[17] for Coulomb fitting. For uranium, a segmented all-electron relativistically contracted (SARC) basis set was used for the orbital basis[18], and a corresponding Coulomb fitting auxiliary basis was automatically generated using the AutoAux procedure.[19] A natural bond orbital (NBO) analysis of the DFT wavefunctions was performed using the NBO code, version 7.0.[20]

References

1. L. R. Avens, C. J. Burns, R. J. Butcher, D. L. Clark, J. C. Gordon, A. R. Schake, B. L. Scott, J. G. Watkin and B. D. Zwick, *Organometallics*, 2000, **19**, 451-457.
2. P. Rungthanaphatsophon, P. Huang and J. R. Walensky, *Organometallics*, 2018, **37**, 1884-1891.
3. C. Ganesamoorthy, S. Loerke, C. Gemel, P. Jerabek, M. Winter, G. Frenking and R. A. Fischer, *Chem. Commun.*, 2013, **49**, 2858-2860.
4. M. Schormann, K. S. Klimek, H. Hatop, S. P. Varkey, H. W. Roesky, C. Lehmann, C. Röpken, R. Herbst-Irmer and M. Noltemeyer, *J. Solid State Chem.*, 2001, **162**, 225-236.
5. P. Jutzi and L. O. Schebaum, *J. Organomet. Chem.*, 2002, **654**, 176-179.
6. R. J. Ward, D. Pividori, A. Carpentier, M. L. Tarlton, S. P. Kelley, L. Maron, K. Meyer and J. R. Walensky, *Organometallics*, 2021, **40**, 1411-1415.
7. Apex4, AXScale, and SAINT, version 2022.1, Bruker AXS, Inc., Madison, WI, 2022.
8. G. Sheldrick, *Acta Cryst. A*, 2015, **71**, 3-8.
9. G. Sheldrick, SHELXS, v.2013-1, 2013.
10. G. Sheldrick, *Acta Cryst. C*, 2015, **71**, 3-8.
11. O. V. Dolomanov, L. J. Bourhis, R. J. Gildea, J. A. K. Howard and H. Puschmann, *J. Appl. Cryst.*, 2009, **42**, 339-341.
12. A. Thorn, B. Dittrich and G. M. Sheldrick, *Acta Cryst. A*, 2012, **68**, 448-451.

13. J. P. Perdew, K. Burke and M. Ernzerhof, *Phys. Rev. Lett.*, 1996, **77**, 3865-3868.
14. F. Neese, F. Wennmohs, U. Becker and C. Riplinger, *J. Chem. Phys.*, 2020, **152**.
15. C. van Wüllen, *J. Chem. Phys.*, 1998, **109**, 392-399.
16. F. Weigend and R. Ahlrichs, *Phys. Chem. Chem. Phys.*, 2005, **7**, 3297-3305.
17. F. Weigend, *Phys. Chem. Chem. Phys.*, 2006, **8**, 1057-1065.
18. D. A. Pantazis and F. Neese, *J. Chem. Theory Comput.*, 2011, **7**, 677-684.
19. G. L. Stoychev, A. A. Auer and F. Neese, *J. Chem. Theory Comput.*, 2017, **13**, 554-562.
20. E. D. Glendening, J. K. Badenhoop, A. E. Reed, J. E. Carpenter, J. A. Bohmann, C. M. Morales, P. Karafiloglou, C. R. Landis, and F. Weinhold, Theoretical Chemistry Institute, University of Wisconsin, Madison, WI (2018)
